# Supplementary material for: Hamster model for post-COVID-19 alveolar regeneration offers an opportunity to understand post-acute sequelae of SARS-CoV-2
Source: Nat Commun. 2023 Jun 5;14:3267. doi: 10.1038/s41467-023-39049-5 (PMC10241385; doi:10.1038/s41467-023-39049-5)
Supplement: Supplementary file 1 — Supplementary information [file 41467_2023_39049_MOESM1_ESM.pdf]

Hamster model for post-COVID-19 alveolar regeneration offers an opportunity to understand post-acute sequelae of SARS-CoV-2

Laura Heydemann[1,7], Małgorzata Ciurkiewicz[1,7] Georg Beythien[1], Kathrin Becker[1], Klaus Schughart[2,3], Stephanie Stanelle-Bertram[4], Berfin Schaumburg[4], Nancy Mounogou-Kouassi[4], Sebastian Beck[4], Martin Zickler[4], Mark Kühnel[5,6], Gülsah Gabriel[4], Andreas Beineke[1], Wolfgang Baumgärtner[1,7] and Federico Armando[1,7]

[1] Department of Pathology, University of Veterinary Medicine, Foundation, Hannover, Germany
[2] Department of Microbiology, Immunology and Biochemistry, University of Tennessee Health Science Center, Memphis, Tennessee, USA,
[3] Institute of Virology Münster, University of Münster, Münster, Germany
[4] Department for Viral Zoonoses-One Health, Leibniz Institute for Virology, Hamburg, Germany
[5] Institute of Pathology, Hannover Medical School (MHH), Hannover, Germany
[6] Member of the German Center for Lung Research (DZL), Biomedical Research in Endstage and Obstructive Lung Disease Hannover (BREATH), Hannover Medical School (MHH), Hannover, Germany
[7] These authors contributed equally

Correspondence and requests for materials should be addressed to Wolfgang Baumgärtner. Wolfgang.baumgaertner@tiho-hannover.de

SUPPLEMENTARY DATA

This file contains:

Supplementary Tables: 3

Supplementary Figures: 29

Supplementary Table 1: Marker gene list of main pulmonary cell populations in the hamster species.

AT2	AT1	ADI	CLUB	CILIATED
Sftpc	*Col12a1*	*S100a10*	*Pigr*	*Ccdc153*
Napsa	*Sema3a*	*Anxa3*	*Ifitm2*	*Caps*
Fabp5	*Dag1*	*S100a6*	*Gss*	*Dynlrb2*
Scd1	*Aqp5*	*Krt7*	*Scgb3a2*	*1700094D03Rik*
Sftpa1	*Gprc5a*	*Krt8*	*Hp*	
Ctsc	*Cav1*	*Dstn*	*Fam216b*	
Lamp3	*Vegfa*	*Anxa1*		
Pgc	*Cav2*	*Tuft1*		
Lgi3	*Itga3*	*Tacstd2*		
Sfta2	*Lama5*	*Klf6*		
Gas6	*Nckap5*	*Lmo7*		
Egfl6	*Abca5*	*Cdkn1a*		
Lcn2	*Itm2a*	*Tp53*		
Atp1a1	*Limd2*	*Tnip3*		
Abca3	*Wsb1*	*Hbegf*		
Sftpd	*Sec14l3*	*Ggh*		
Slc34a2	*Prdx6*	*Steap4*		
Serpine2	*Mfge8*	*Zfp36*		
	Ccnd2	*Junb*		
	Timp3	*Jun*		
		Fos		
		CRYAB		
		Ndnf		
		Timp2		
		Emp2		
		Sox4		
		Wwtr1		
		Sparc		

Abbreviations: alveolar pneumocytes type 2 (AT2), alveolar pneumocytes type 1 (AT1), alveolar differentiation intermediate cells (ADI), club cells (CLUB), ciliated cells (CILIATED)

Supplementary Table 2: Primary antibodies, visualization method, dilution, clonality and host species, secondary antibody as well as positive controls used for immunohistochemical investigations.

Primary antibody	Visualization method	Dilution	Clonality, host species	Secondary antibody (1:200)	Positive control
CK8 (Invitrogen, PA5-29607)	EnVision	1 : 250	Polyclonal, rabbit	/	Airways (Hu, Ms, Hm internal control)
CK14 (Invitrogen, PA5-16722)	ABC	1 : 500	Polyclonal, rabbit	GAR-b	Airways (Hu, Ms, Hm internal control)
SCGB1A1 (Proteintec, 10490-1-AP)	ABC	1 : 200	Polyclonal, rabbit	GAR-b	Airways (Hu, Ms, Hm internal control)
proSP-C (MEMD Millipore, AB3786)	EnVision	1 : 1000	Polyclonal, rabbit	/	Alveolar AT2 (Hu, Ms, Hm internal control)
IBA-1 (FUJIFILM Wako Pure Chemical Corporation, 019–19741)	ABC	1 : 500	Polyclonal, rabbit	GAR-b	SARS-CoV-2 Infected lung, 6 dpi (Hm internal control)
SARS CoV-2-NP (Sinobiological, 40143-MM05)	EnVision	1 : 16000	Monoclonal, mouse, clone 5	GAM-b	SARS-CoV-2 Infected lung, 3 dpi (Hm internal control)
α-SMA (Dako, GA611)	ABC	1: 500	Monoclonal, mouse, Clone 1A4	GAM-b	Airways and vessels (Hu, Ms, Hm internal control)
CD-204 (Abnova Corporation, MAB1710)	ABC	1:1000	Monoclonal, mouse, clone SRA-E5	GAM-b	SARS-CoV-2 Infected lung, 6 dpi (Hm internal control)

Abbreviations: α-SMA: alpha smooth muscle actin; ABC: Avidin-biotin-complex; CK8: cytokeratin 8; CK14: cytokeratin 14; GAR-b: goat anti rabbit-biotinylated; GAM-b: goat anti mouse-biotinylated; Hm: hamster; Hu: human; IBA-1: ionized calcium-binding adapter molecule 1; Ms: mouse; SARS-CoV-2 NP: severe acute respiratory syndrome coronavirus-2 nucleo protein; SCGB1A1: secretoglobin 1A1; proSP-c: pro surfactant protein C;

Supplementary Table 3: Primary antibodies, dilution, clonality and host species as well as secondary antibody used for immunofluorescence investigations.

Primary antibody	Dilution	Clonality, host species	Secondary antibody (1:200)
CK8 (Invitrogen, PA5-29607)	1 : 500	Polyclonal, rabbit	GAR Cy2/ GAR Cy3
CK8-FITC conjugated (abcam, ab192467)	1 : 200	Monoclonal, rabbit, clone EP1628Y	/
CK14 (Invitrogen, PA5-16722)	1 : 500	Polyclonal, rabbit	GAR Cy2
CK14 (Invitrogen, MA5-11599)	1 : 500	Monoclonal, mouse, clone LL002	GAM Cy2/ GAMCy3
proSP-C (MEMD Millipore, AB3786)	1 : 1000	Polyclonal, rabbit	GAR Cy3
SCGB1A1 (Proteintec, 10490-1-AP)	1 : 200	Polyclonal, rabbit	GAR Cy3
TP53 (Novusbio, NBP2-29453)	1 : 100	Monoclonal, mouse, clone BP53-12	GAM Cy3
ΔNp63 (Cell signalling, #67825S)	1 : 800	Monoclonal, rabbit, clone E6Q30	GAR Cy3
CK5-FITC conjugated (Abcam, ab-193894)	1 : 200	Monoclonal, rabbit, clone EP1601Y	/

Abbreviations:Cy2: cyanin 2 conjuagated; Cy3: cyianin 3 conjugated; CK8: cytokeratin 8; CK14: cytokeratin 14; CK5: cytokeratin 5; GAR-b: goat anti rabbit-biotinylated; GAM-b: goat anti mouse-biotinylated;

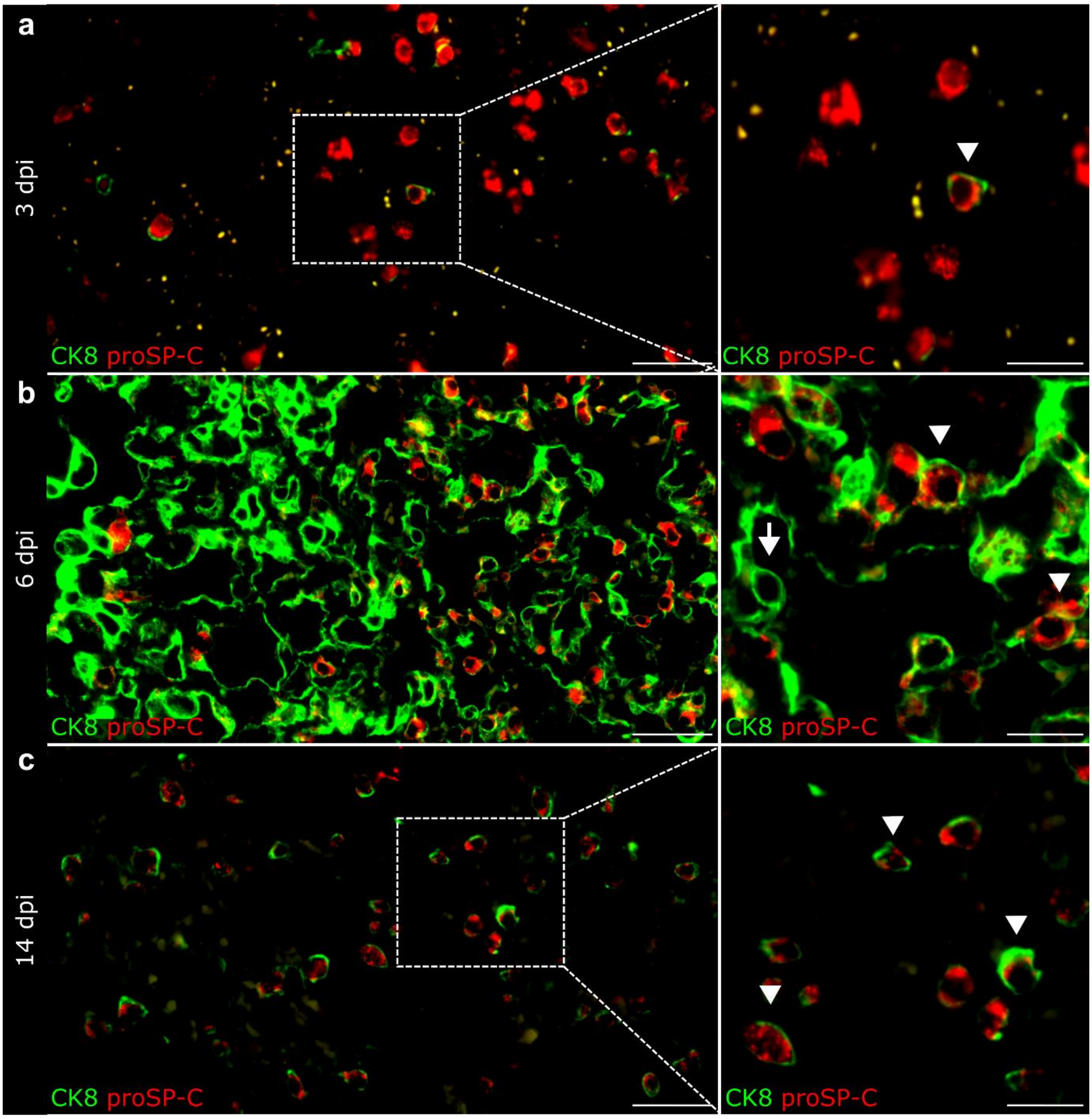

Supplementary figure 1: Alveolar pneumocytes type 2 (AT2) - alveolar differentiation intermediate (ADI) cell trajectory in SARS-CoV-2 infected hamsters at different time points post infection.

Representative double immunofluorescence images of alveoli in a SARS-CoV-2 infected hamsters at 3 (A), 6 (B) and 14 (C) days post infection (dpi). Cells are labelled with CK8 (green) and proSP-C (red). For each time point, an overview and higher magnification of the area delineated by the rectangle are shown. **A** At 3 dpi, there are numerous, round, proSP-C^{+}CK8^{-} AT2 cells and rare, round, proSP-C^{+}CK8^{+} ADI cells (arrowhead). **B** Alveolar proliferation at 6 dpi contain numerous proSP-C^{-}CK8^{+}ADI cells, some showing hypertrophy and elongated cytoplasmic processes (arrow). There are single proSP-C^{+}CK8^{+} cells with a round morphology (arrowheads). **C** At 14 dpi, there are numerous round, proSP-C^{+}CK8^{+} ADI cells (arrowheads) and rare round proSP-C^{+}CK8^{-} AT2 cells. Scale bars: 50 µm (left panel) and 20 µm (right panel).

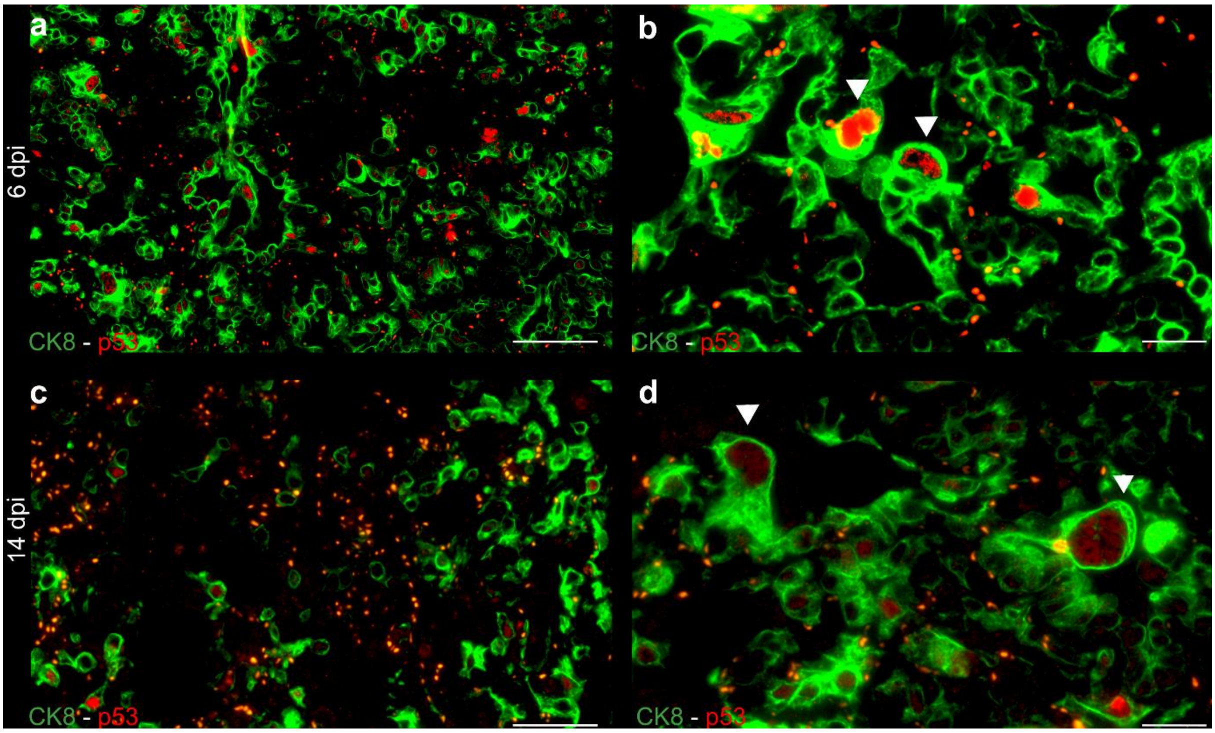

Supplementary figure 2: Alveolar differentiation intermediate (ADI) cells exhibit cell cycle arrest in SARS-CoV-2 infected hamsters at different time points post infection.

Representative double immunofluorescence images of alveoli in SARS-CoV-2 infected hamsters at 6 (A, B) and 14 (C, D) days post infection (dpi). Cells are labelled with CK8 (green) and TP53 (red). **A, B** Overview and high magnification of proliferation focus at 6 dpi showing numerous $CK8^{+}$ ADI expressing nuclear TP53. The high magnification shows polygonal, large, bizarre $TP53^{+}$ ADI cells (arrowheads). **C** Overview of morphologically normal alveoli at 14 dpi showing numerous $TP53^{+}$ ADI cells with a round morphology. **D** Large, bizarre $TP53^{+}$ ADI cells (arrowheads) within residual alveolar lesions at 14 dpi. Scale bars: 50 µm and 20 µm (b,d).

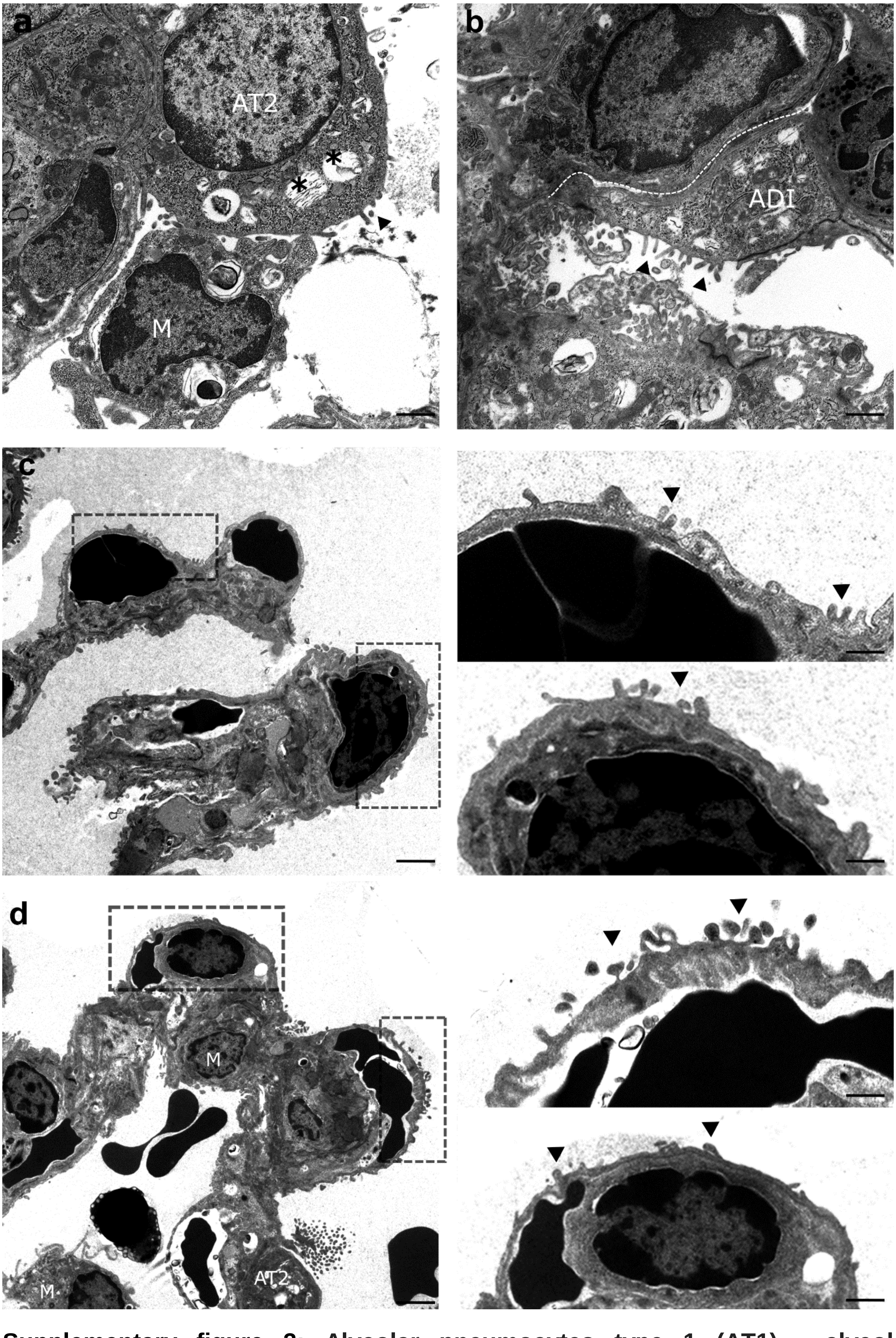

Supplementary figure 3: **Alveolar pneumocytes type 1 (AT1) - alveolar differentiation intermediate (ADI) cells trajectory in SARS-CoV-2 infected hamsters.**

A Transmission electron microscopy (TEM) micrograph of normal alveolar cells showing a round cell (AT2) with apico-basal polarity, apical microvilli (arrowhead), moderately electron-dense cytoplasm, rich in rough endoplasmic reticulum and free ribosomes as well as numerous membrane-bound vesicles containing multiple concentric membrane layers (multi-lamellar bodies, asterisks). On the left, a macrophage with intracytoplasmic multi-lamellar bodies is also seen (M). **B** Representative micrograph showing alveoli of a SARS-CoV-2 infected hamster at 6 dpi. In the center, a stretching cell (ADI) showing AT2 features like ribosome-rich cytoplasm and microvilli (arrowheads) on the cell surface is seen. The dotted line indicates the basal contour of the cell, highlighting the elongated shape. **C, D** Representative micrograph showing alveoli of SARS-CoV-2 infected hamsters at 6 dpi. Overviews and higher magnification of the areas delineated by rectangles are shown. Alveolar septae are covered by delicate, elongated cells, separated by a thin basement membrane from capillaries containing erythrocytes. Macrophages (M) as well as an AT2 cell (AT2) are also seen. Rectangles and high magnification show cells with flattened and elongated morphology of AT1 cells but also retained characteristics of AT2 cells, such as apical microvilli (arrowheads), indicative of a transitional state. Scale bars: 1000 nm (a), 500 nm, 2000 (overview in c, d), 500 nm (high magnifications in c, d).

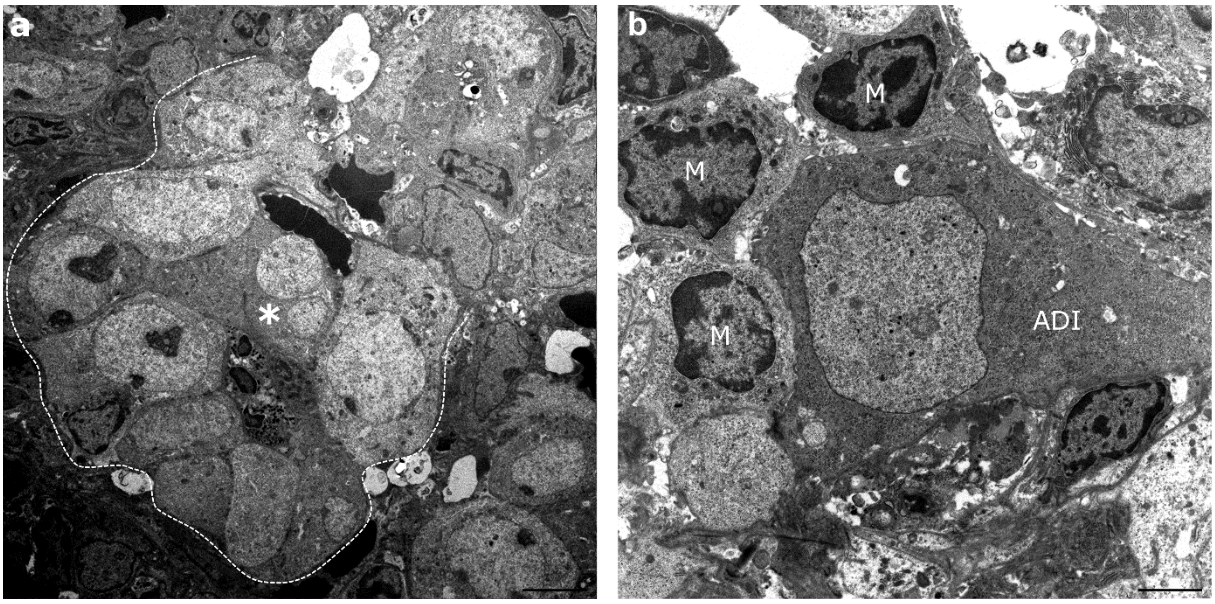

Supplementary figure 4: Epithelial proliferates and in SARS-CoV-2 infected hamsters.

A Transmission electron microscopy (TEM) micrograph of an epithelial proliferation focus from a SARS-CoV-2 infected hamster at 6 days post infection (dpi). A string of proliferating, polygonal to columnar epithelial cells forming a tubule-like structure (dotted line) is shown. The cells show hypertrophy, numerous prominent mitochondria and irregularly clumped chromatin. A bizarre cell with two unevenly large nuclei is also present (asterisk). **B** Micrograph of an ADI cell within a proliferation focus from a SARS-CoV-2 infected hamster at 6 dpi. A triangular, hypertrophic ADI cell surrounded by multiple macrophages (M) is shown. Scale bars: 500 nm.

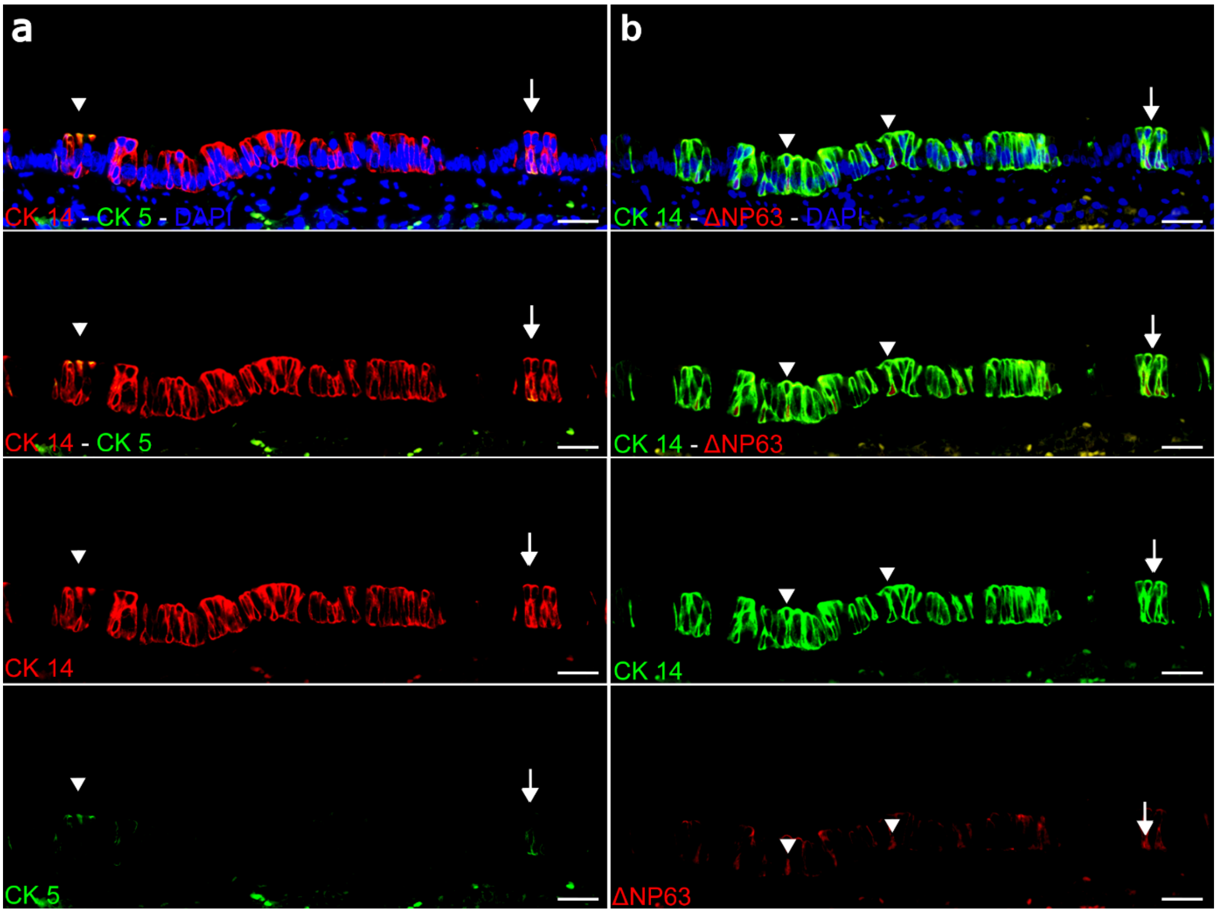

Supplementary figure 5: basal cells in the airways of hamsters.

A Representative images of double immunofluorescence for CK5 (green) and CK14 (red) in a hamster airway, showing numerous $CK14^{+}CK5^{-}$ cells and occasional double labelled $CK14^{+}CK5^{+}$ cells (arrowheads). **B** Representative image of double immunofluorescence for CK14 (green) and ΔNP63 (red) in a hamster airway, taken at the same location as the images in a. There are numerous $CK14^{+}\Delta Np63^{-}$ cells and fewer $CK14^{+}\Delta Np63^{+}$ cells (arrowheads). Cells indicated by an arrow in A and B are considered $CK5^{+}CK14^{+}\Delta Np63^{+}$. Scale bars: 25 µm.

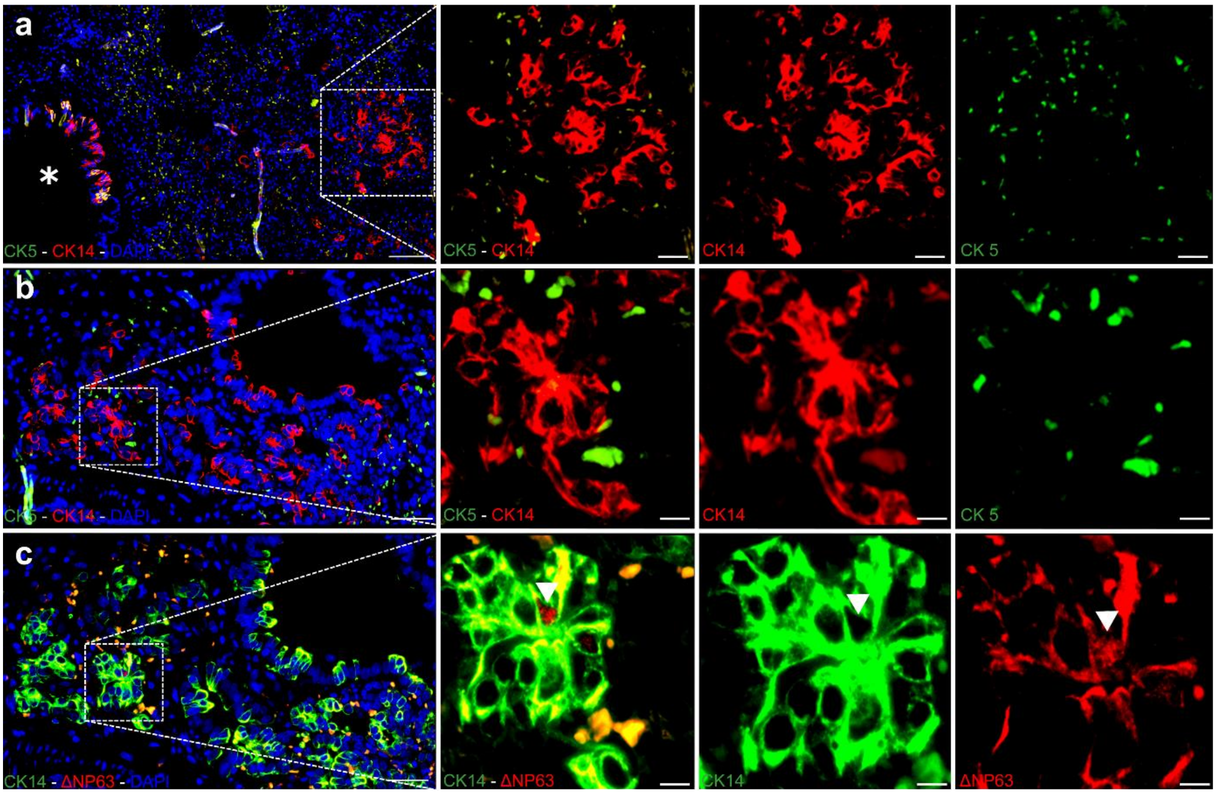

Supplementary figure 6: CK14⁺ and CK14⁺ΔNp63⁺ basal cells take part in alveolar proliferation in SARS-CoV-2 infected hamsters.

A Representative image of double immunofluorescence for CK5 (green) and CK14 (red) in a peribronchiolar proliferation area in a SARS-CoV-2 infected hamster at 6 dpi. An overview and higher magnification of the area delineated by a rectangle are shown. The asterisk indicates a bronchiole containing $CK5^{+}CK14^{+}$ basal cells. Alveolar proliferation foci are composed of $CK5^{-}CK14^{+}$ basal cells. **B**, **C** Representative images of double immunofluorescence for CK5 (green) and CK14 (red) as well as CK14 (green) and ΔNP63 (red), respectively. Pictures are taken from the same peribronchiolar proliferation area in a SARS-CoV-2 infected hamster at 6 dpi. Overviews and higher magnification of the area delineated by rectangles are shown. Alveolar proliferation foci are mainly composed of $CK5^{-}CK14^{+}\Delta NP63^{-}$ and rare $CK5^{-}CK14^{+}\Delta NP63^{+}$ basal cells (arrowhead). Scale bars: 50 µm (overviews), 25 µm (high magnifications in a), 10 µm (high magnifications in b, c).

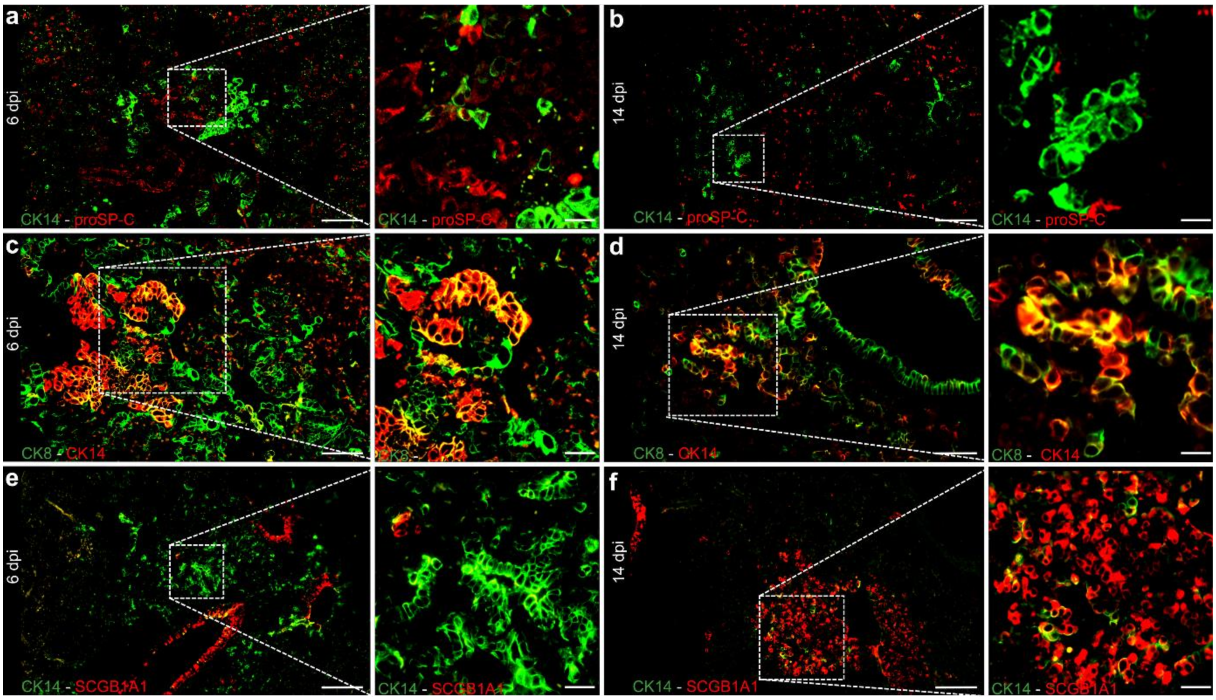

Supplementary figure 7: Airway basal cells in alveolar proliferates of SARS-CoV-2 infected hamsters at different time points.

A, B Representative images of double immunofluorescence for CK14 (green) and proSP-C (red) in peribronchiolar proliferation areas in SARS-CoV-2 infected hamsters at 6 and 14 dpi, respectively. An overview and higher magnification of the areas delineated by rectangles are shown. **A** $CK14^+$ basal cells in the alveoli. Single cells are $CK14^+proSP\text{-}C^+$, indicating that airway progenitors differentiate into $proSP\text{-}C^+$ AT2 cells at 6 dpi. **B** $CK14^+$ basal cells populating the alveolar proliferation foci without transdifferentiating in AT2 cells at 14 dpi. **C, D** Representative images of double immunofluorescence for CK14 (red) and CK8 (green) in peribronchiolar proliferation areas in SARS-CoV-2 infected hamsters at 6 and 14 dpi, respectively. An overview and higher magnification of the area delineated by a rectangle are shown. **C** Transition from $CK14^+$ airway basal cells forming a pod, to double labeled $CK14^+CK8^+$ cells differentiating into $CK14^-CK8^+$, elongated ADI cells at 6 dpi. **D** $CK14^+$ cells with airway-like morphology, populating the alveolar proliferation foci without transdifferentiating in elongated ADI cells at 14 dpi. **E, F** Representative images of double immunofluorescence for CK14 (green) and SCGB1A1 (red) in peribronchiolar proliferation areas in SARS-CoV-2 infected hamsters at 6 and 14 dpi, respectively. An overview and higher magnification of the area delineated by a rectangle are shown. **E** $CK14^+$ cells populating the alveolar proliferatio focis without transdifferentiating in $SCGB1A1^+$ club cells at 6 dpi. **F** $CK14^+$ basal cells in the alveoli. Numerous cells are $CK14^+SCGB1A1^+$ indicating that airway progenitors differentiate into $SCGB1A1^+$ club cells at 14 dpi. Scale bars: 50 µm (overviews), 25 µm (high magnifications).

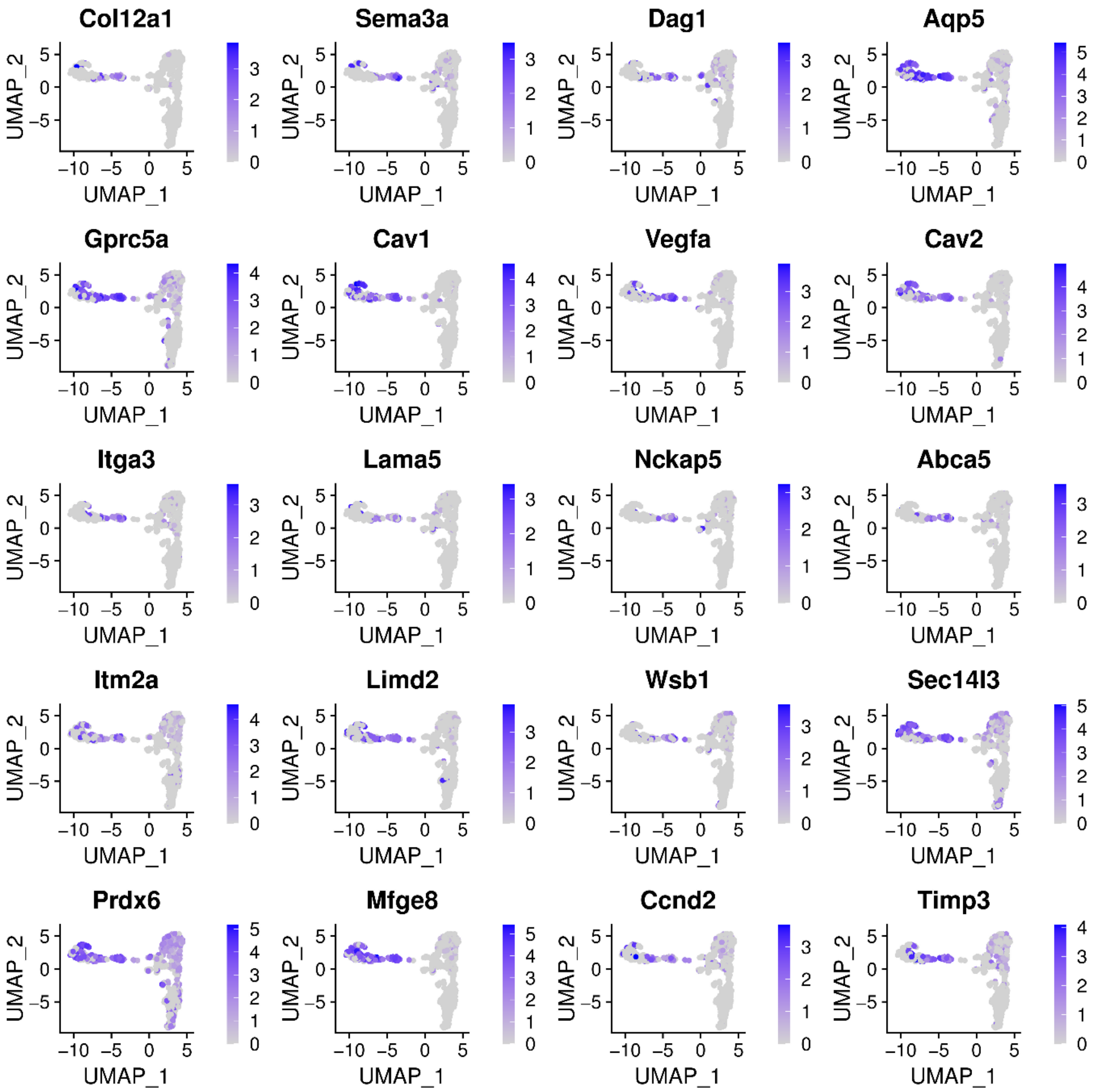

Supplementary figure 8: Feature plots for alveolar type 1 (AT1) cell marker genes in SARS-CoV-2 infected hamsters. Results for AT1 cell genes at 5 dpi. All genes were plotted individually. For AT1 cell marker gene list, see Supplementary Table 1.

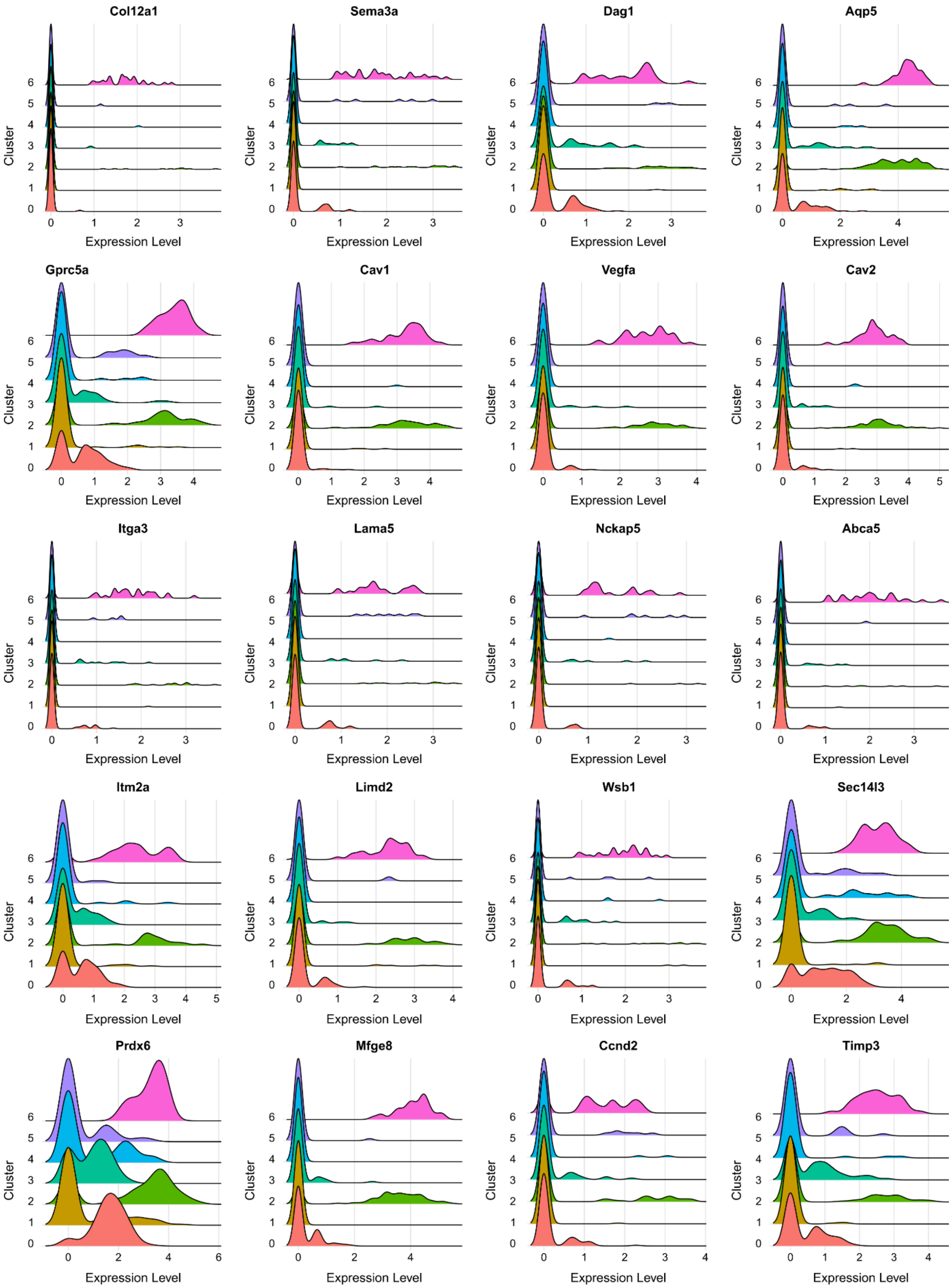

Supplementary figure 9: Ridge plots for alveolar type 1 (AT1) cell marker genes in SARS-CoV-2 infected hamsters. Results for AT1 cell genes at 5 dpi. All genes were plotted individually. For AT1 cell marker gene list, see Supplementary Table 1.

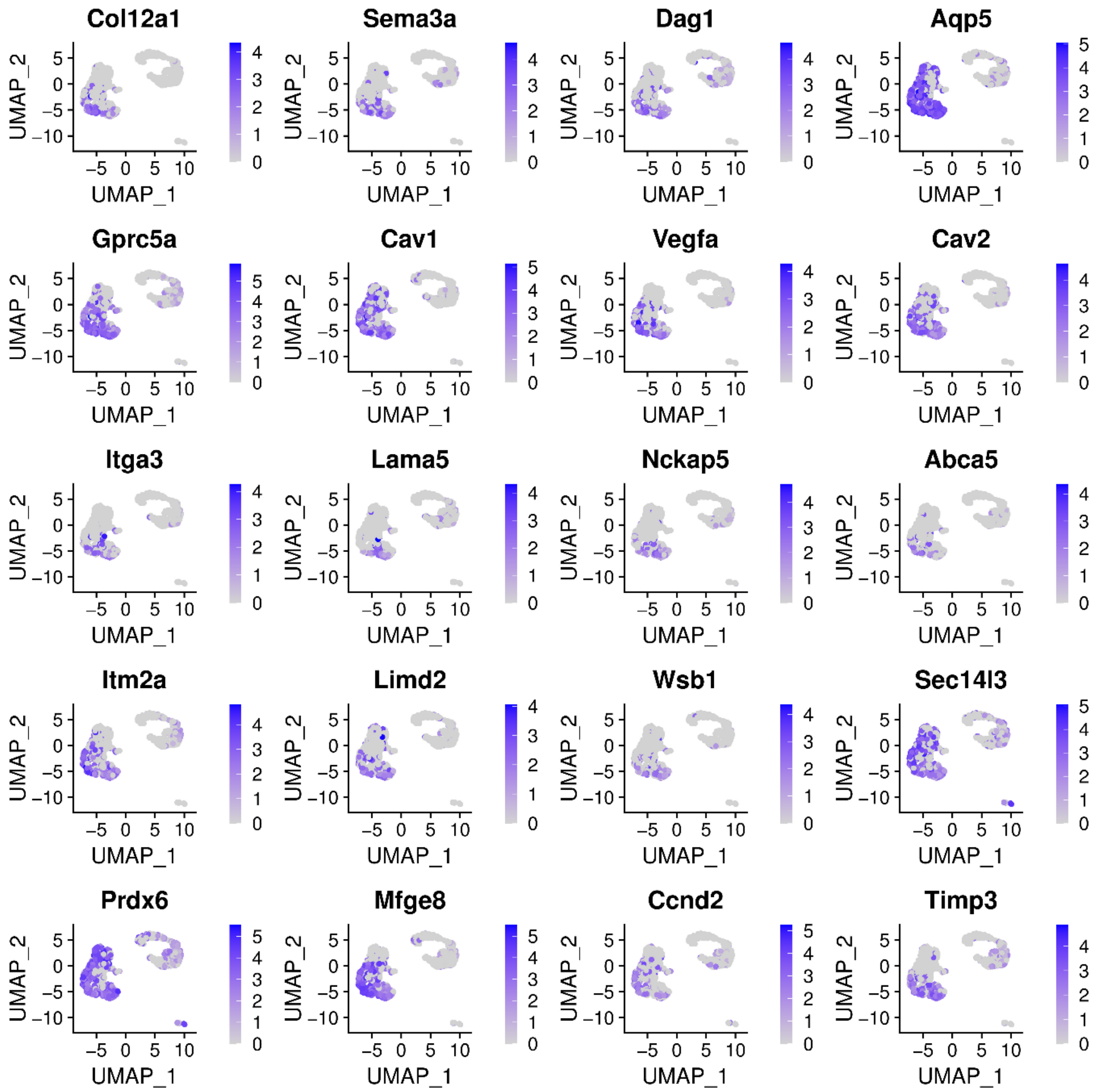

Supplementary figure 10: Feature plots for alveolar type 1 (AT1) cell marker genes in SARS-CoV-2 infected hamsters. Results for AT1 cell genes at 14 dpi. All genes were plotted individually. For AT1 cell marker gene list, see Supplementary Table 1.

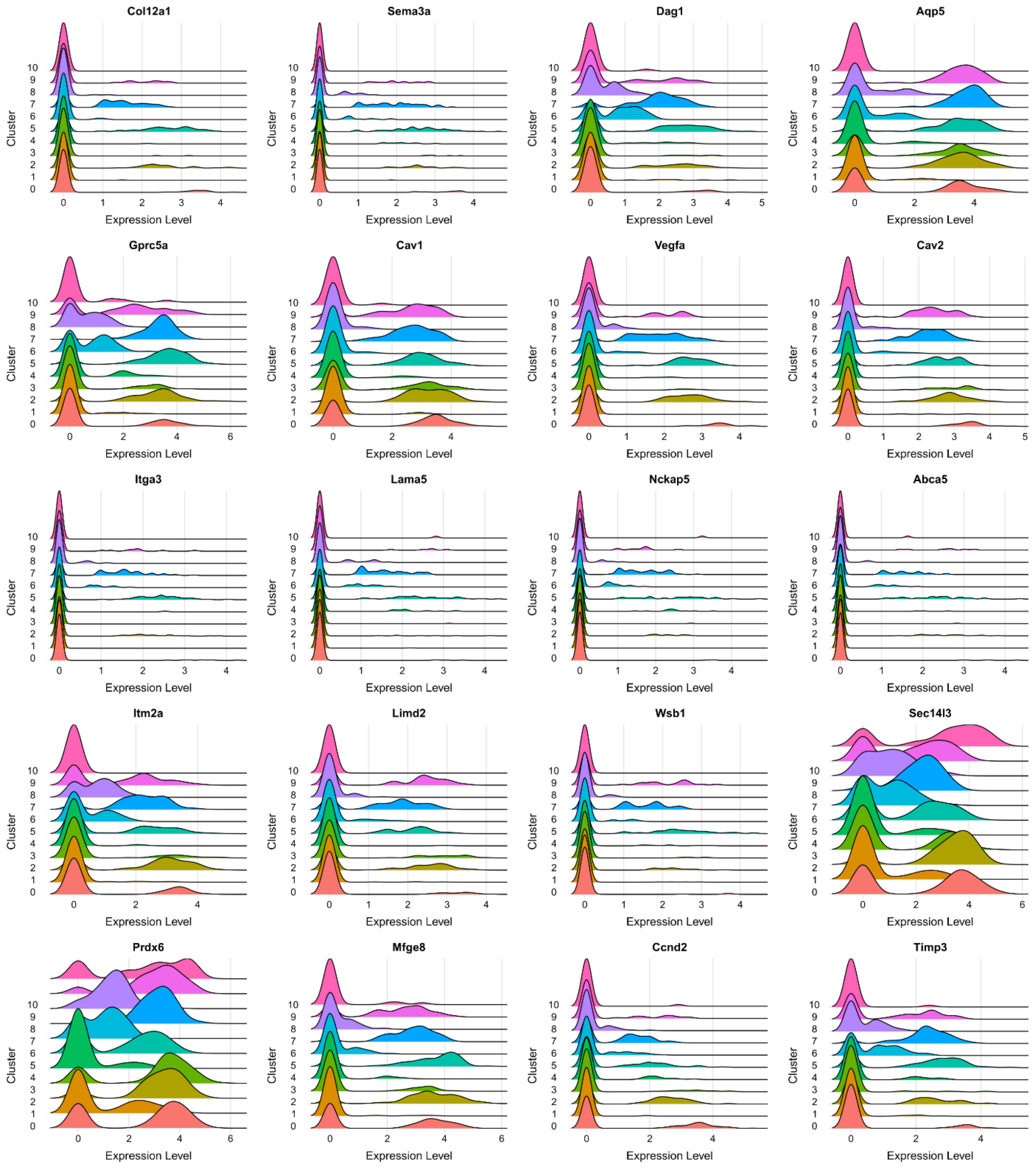

Supplementary figure 11: Ridge plots for alveolar type 1 (AT1) cell marker genes in SARS-CoV-2 infected hamsters. Results for AT1 cell genes at 14 dpi. All genes were plotted individually. For AT1 cell marker gene list, see Supplementary Table 1.

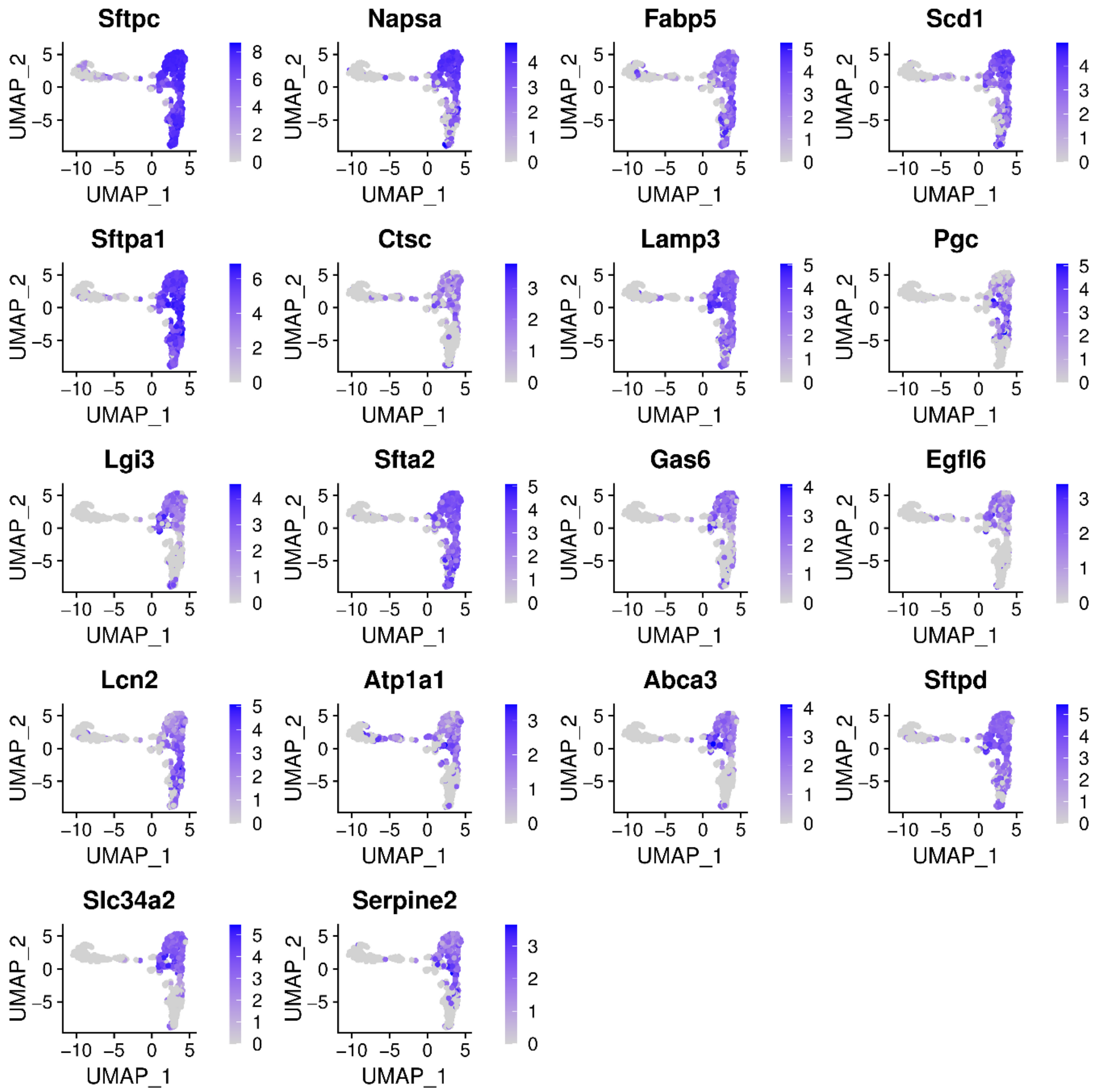

Supplementary figure 12: Feature plots for alveolar type 2 (AT2) cell marker genes in SARS-CoV-2 infected hamsters. Results for AT2 cell genes at 5 dpi. All genes were plotted individually. For AT2 cell marker gene list, see Supplementary Table 1.

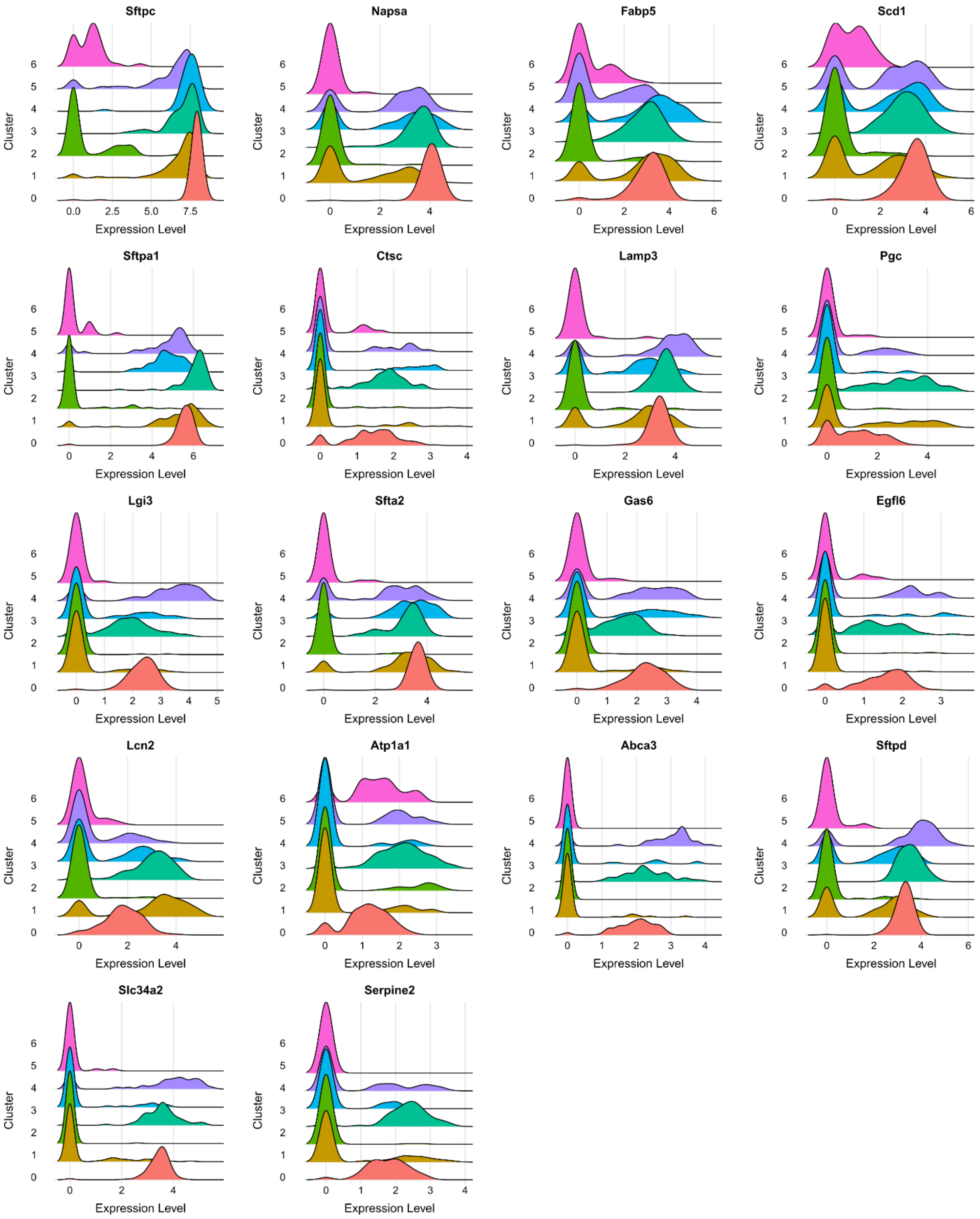

Supplementary figure 13: Ridge plots for alveolar type 2 (AT2) cell marker genes in SARS-CoV-2 infected hamsters. Results for AT2 cell genes at 5 dpi. All genes were plotted individually. For AT2 cell marker gene list, see Supplementary Table 1

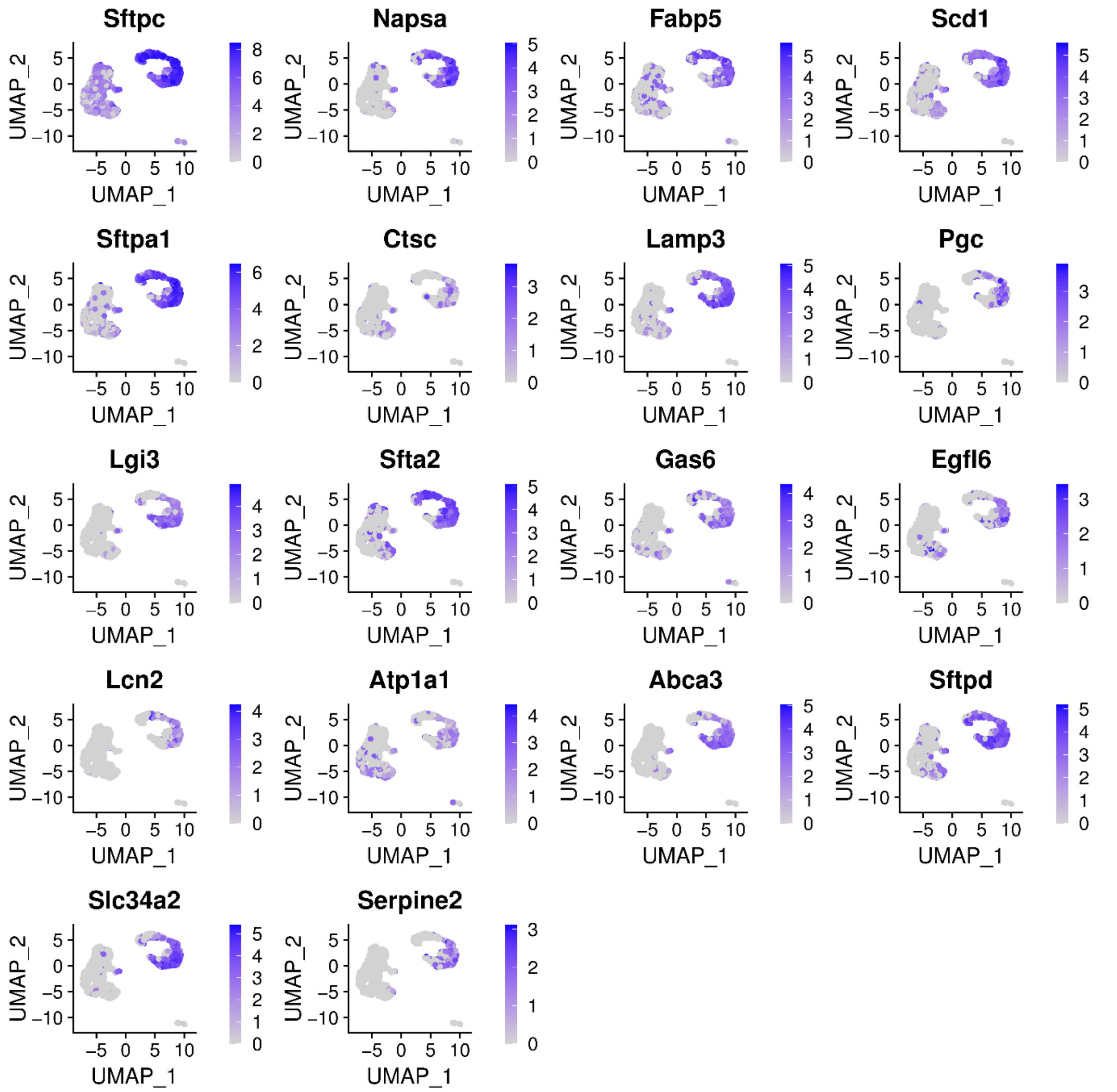

Supplementary figure 14: Feature plots for alveolar type 2 (AT2) cell marker genes in SARS-CoV-2 infected hamsters. Results for AT2 cell genes at 14 dpi. All genes were plotted individually. For AT2 cell marker gene list, see Supplementary Table 1.

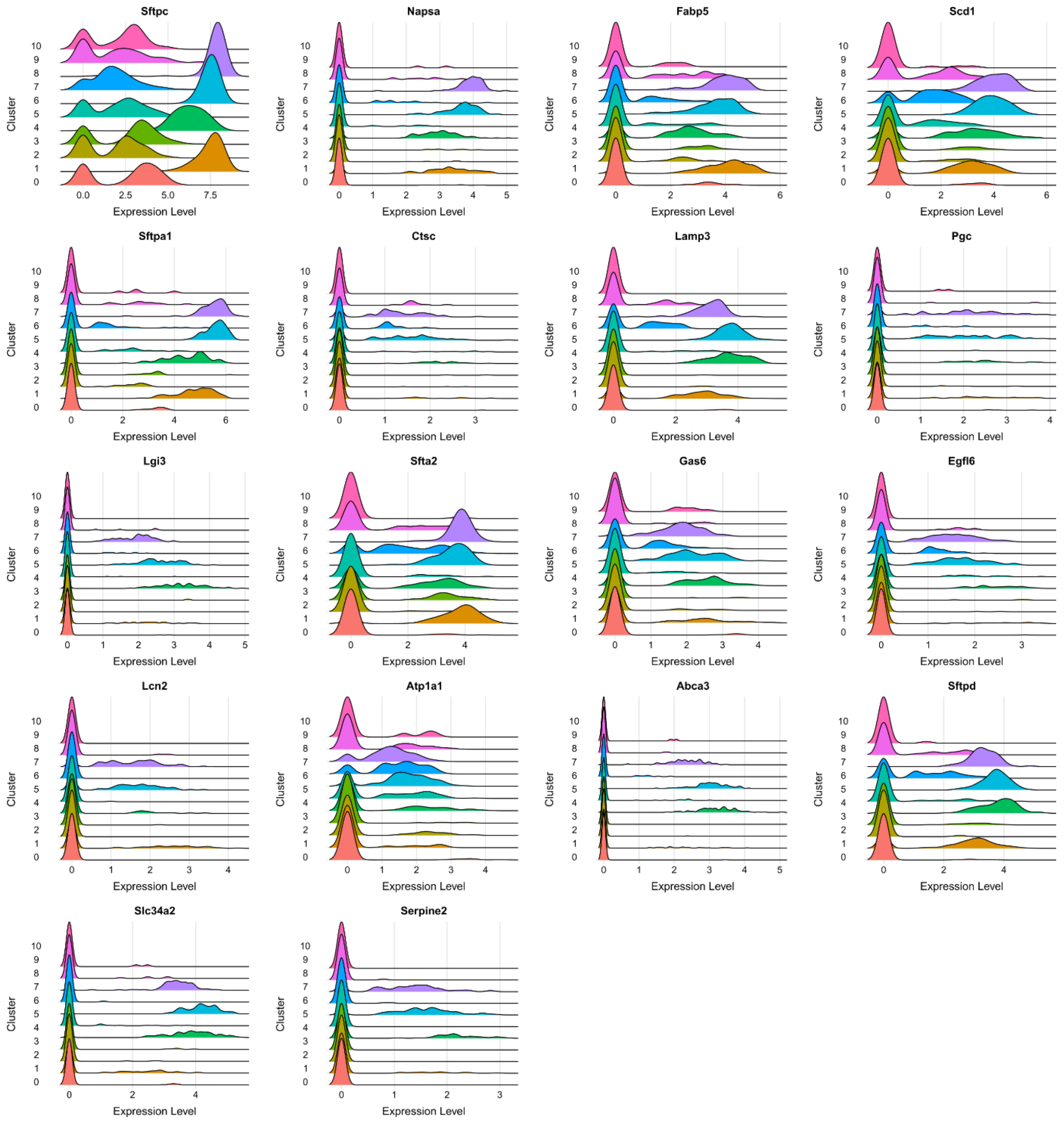

Supplementary figure 15: Ridge plots for alveolar type 2 (AT2) cell marker genes in SARS-CoV-2 infected hamsters. Results for AT2 cell genes at 14 dpi. All genes were plotted individually. For AT2 cell marker gene list, see Supplementary Table 1.

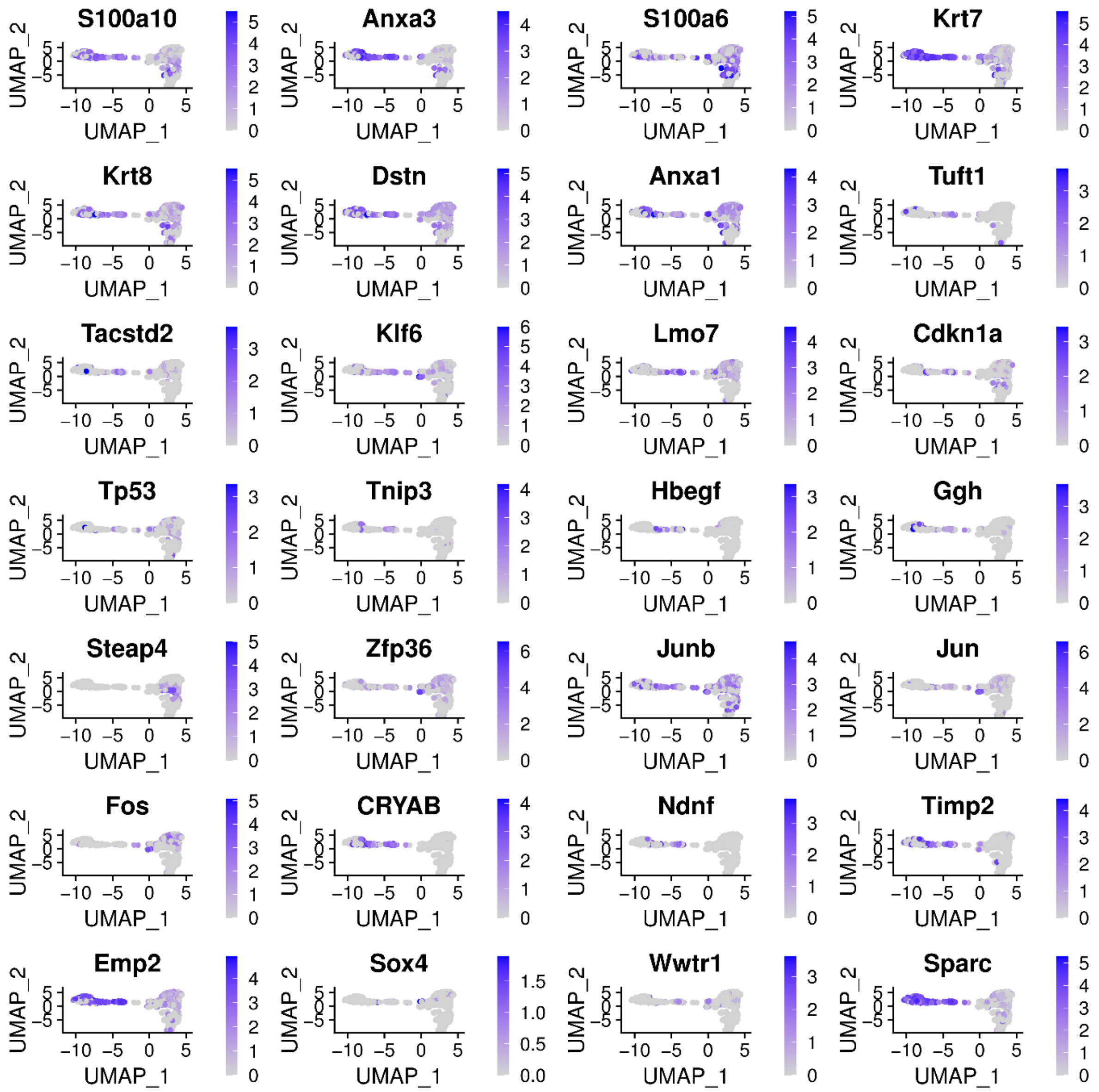

Supplementary figure 16: Feature plots for alveolar differentiation intermediate (ADI) cell marker genes in SARS-CoV-2 infected hamsters. Results for ADI cell genes at 5 dpi. All genes were plotted individually.For ADI cell marker gene list, see Supplementary Table 1.

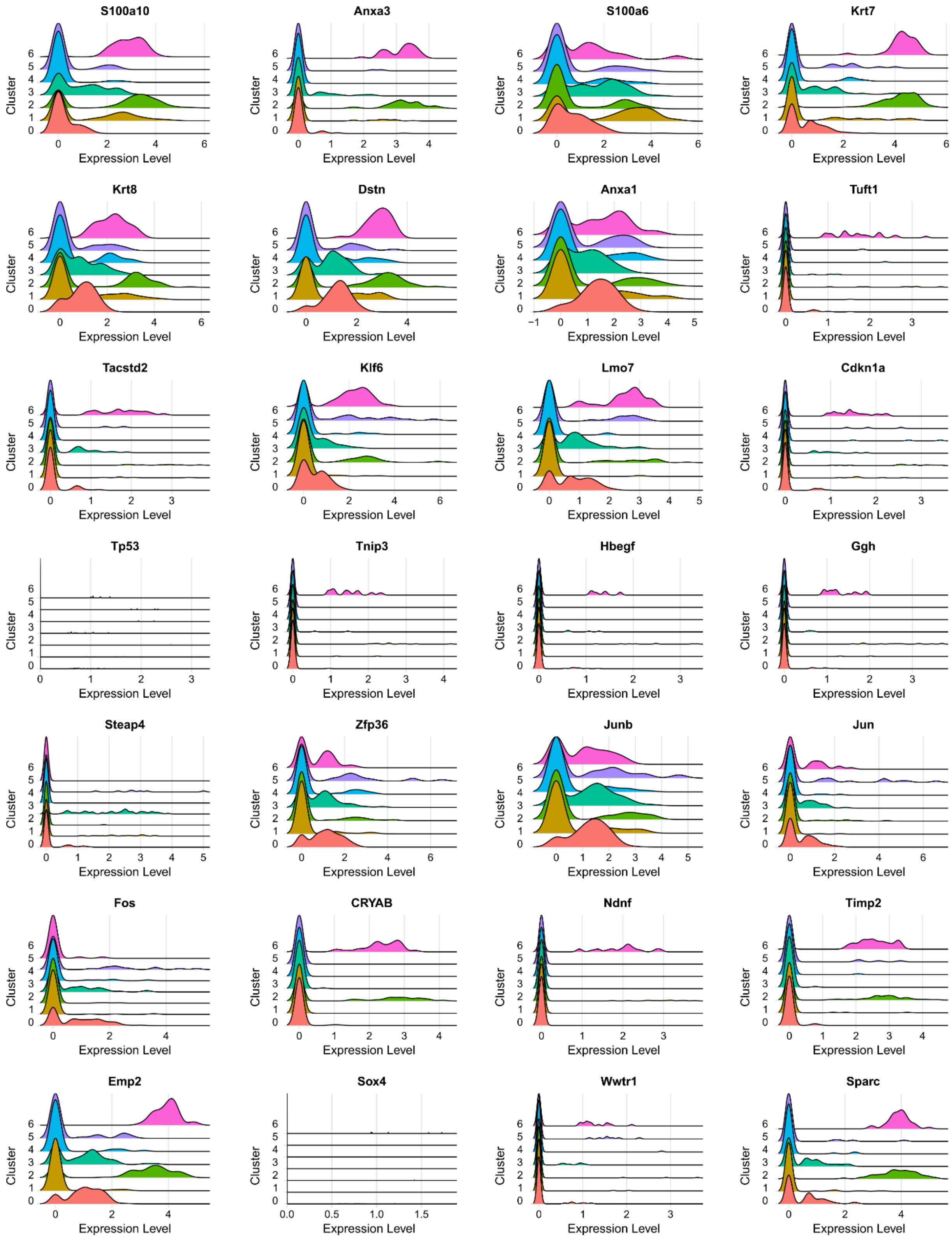

Supplementary figure 17: Ridge plots for alveolar differentiation intermediate (ADI) cell marker genes in SARS-CoV-2 infected hamsters. Results for ADI cell genes at 5 dpi. All genes were plotted individually.For ADI cell marker gene list, see Supplementary Table 1.

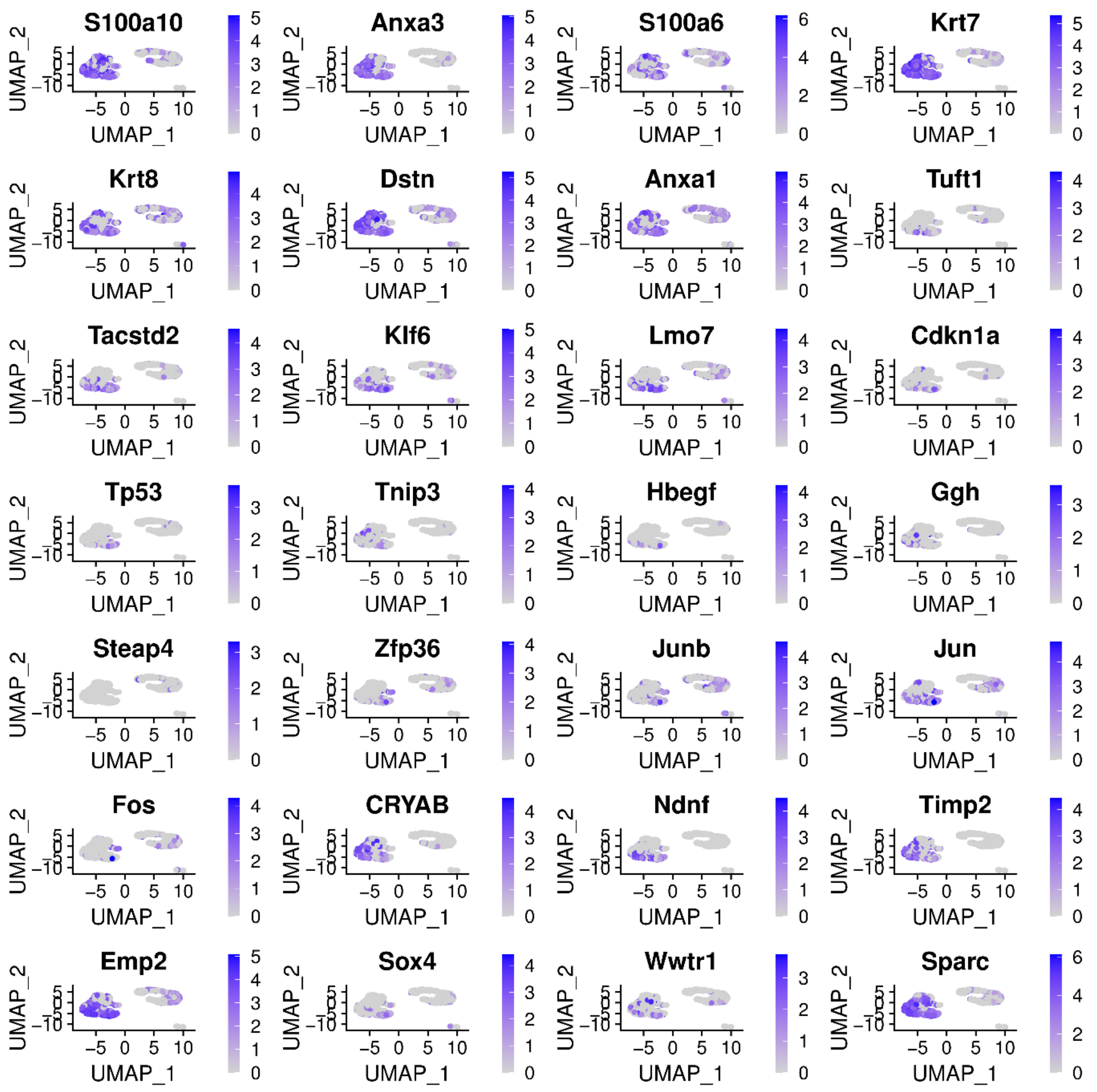

Supplementary figure 18: Feature plots for alveolar differentiation intermediate (ADI) cell marker genes in SARS-CoV-2 infected hamsters. Results for ADI cell genes at 14 dpi. All genes were plotted individually. For ADI cell marker gene list, see Supplementary Table 1.

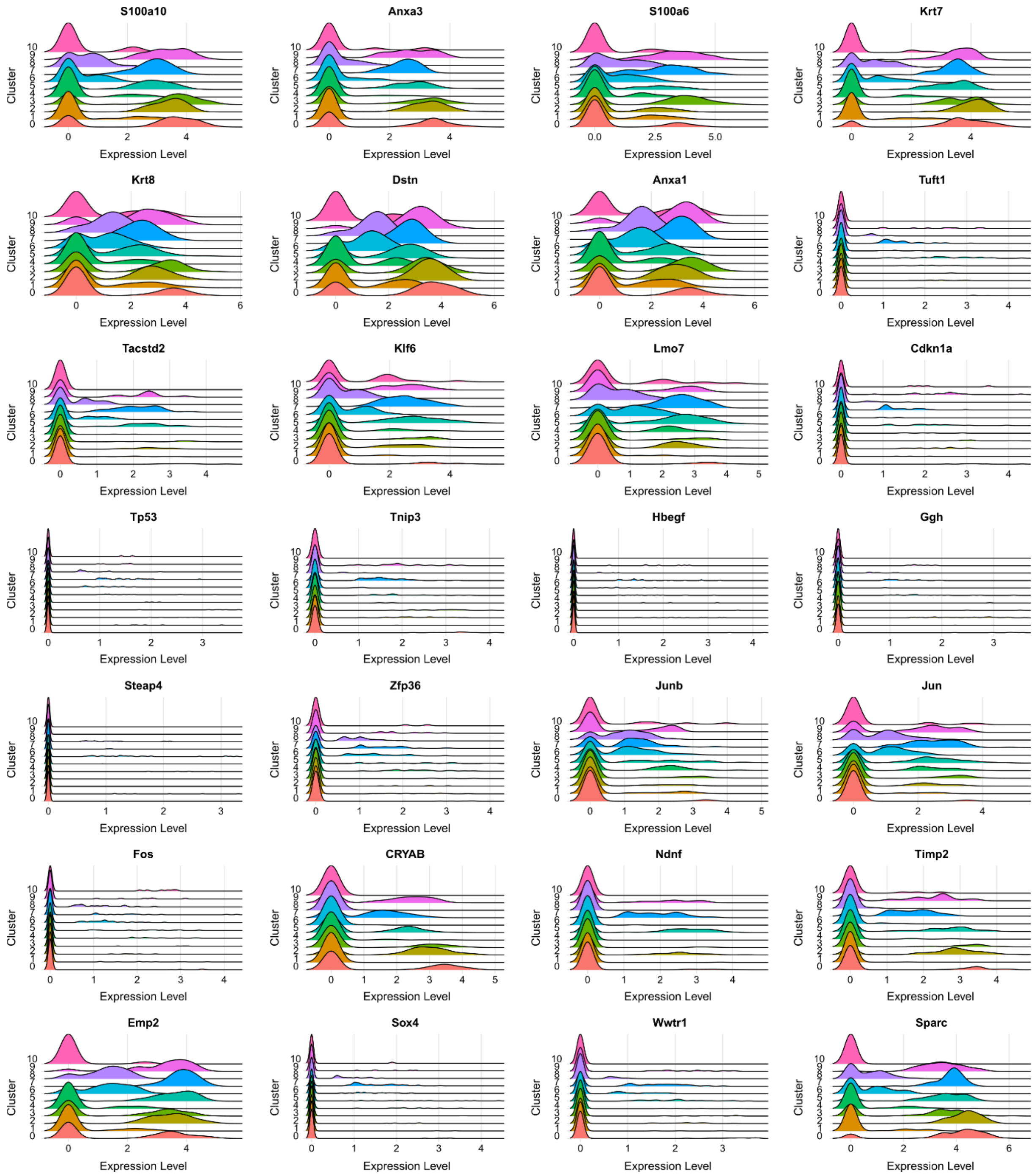

Supplementary figure 19: Ridge plots for alveolar differentiation intermediate (ADI) cell marker genes in SARS-CoV-2 infected hamsters. Results for ADI cell genes at 14 dpi. All genes were plotted individually.For ADI cell marker gene list, see Supplementary Table 1.

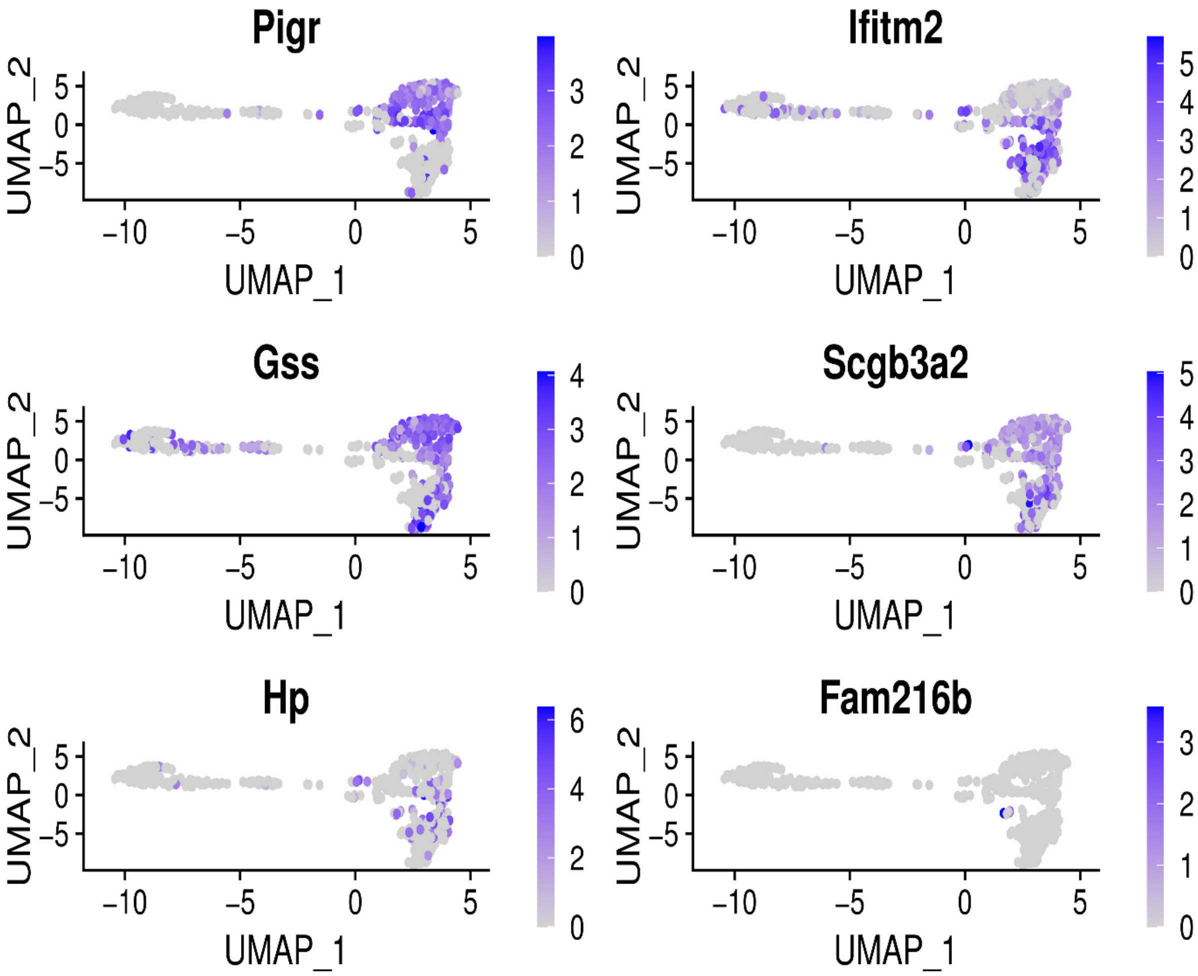

Supplementary figure 20: Feature plots for club cell marker genes in SARS-CoV-2 infected hamsters. Results for club cell genes at 5 dpi. All genes were plotted individually. For club cell marker gene list, see Supplementary Table 1.

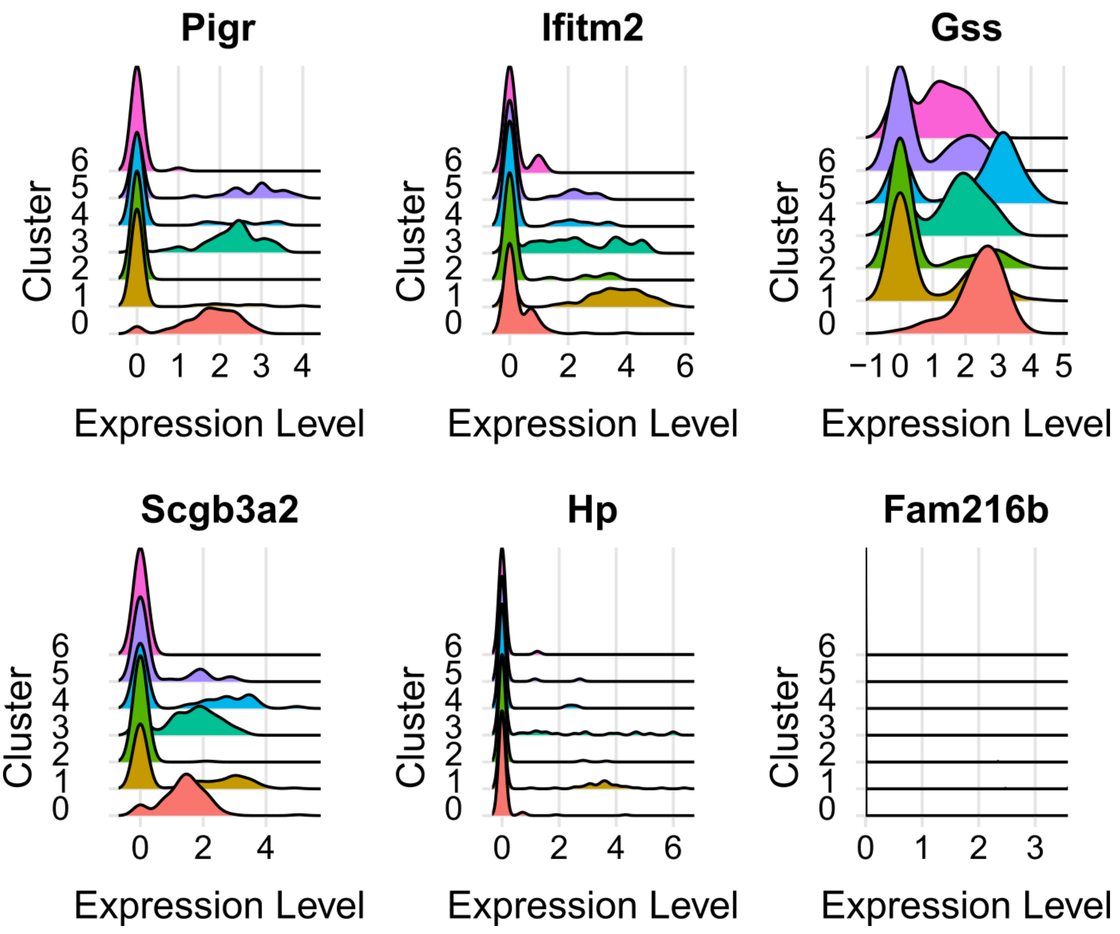

Supplementary figure 21: Ridge plots for club cell marker genes in SARS-CoV-2 infected hamsters. Results for club cell genes at 5 dpi. All genes were plotted individually. For club cell marker gene list, see Supplementary Table 1.

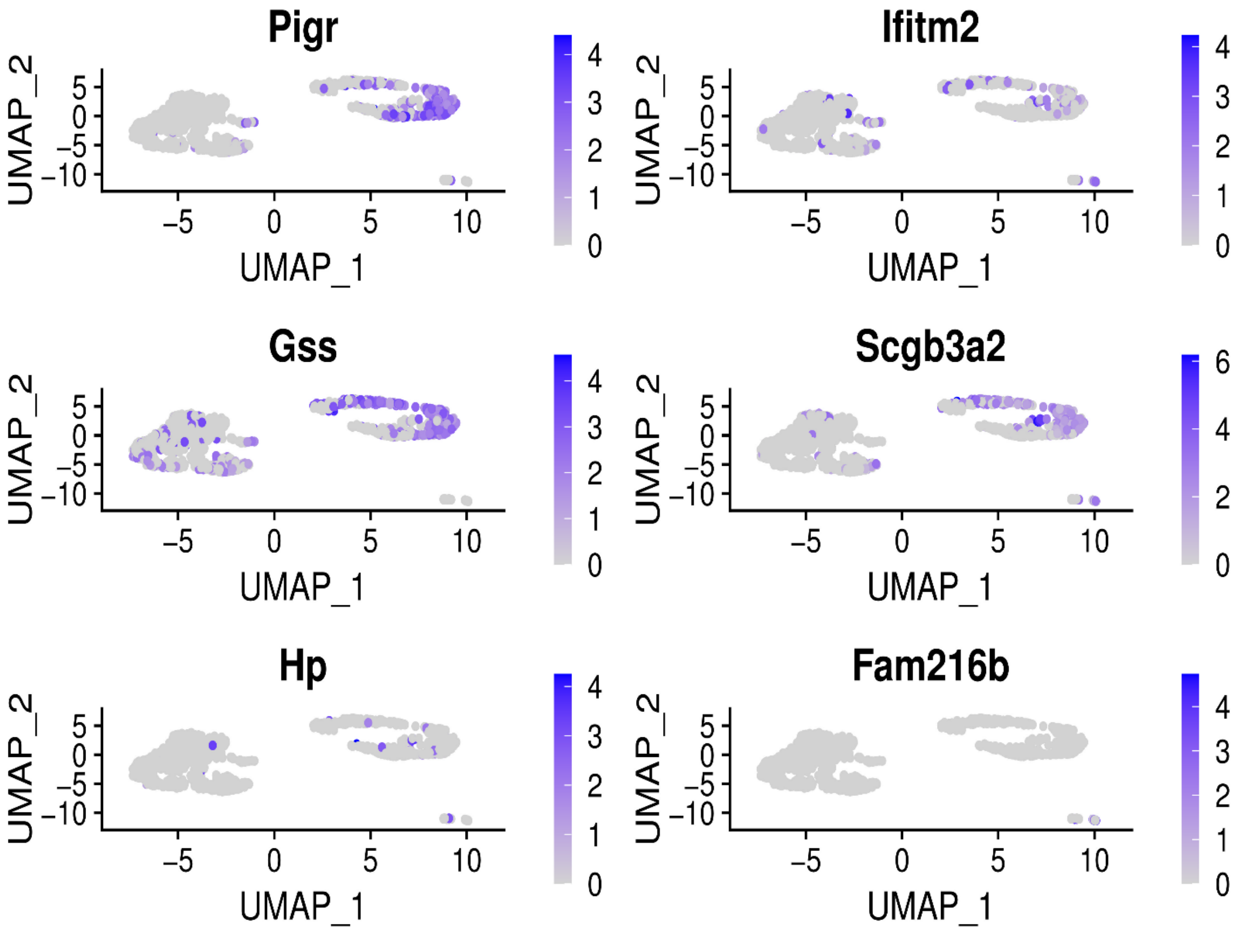

Supplementary figure 22: Feature plots for club cell marker genes in SARS-CoV-2 infected hamsters. Results for club cell genes at 14 dpi. All genes were plotted individually. For club cell marker gene list, see Supplementary Table 1.

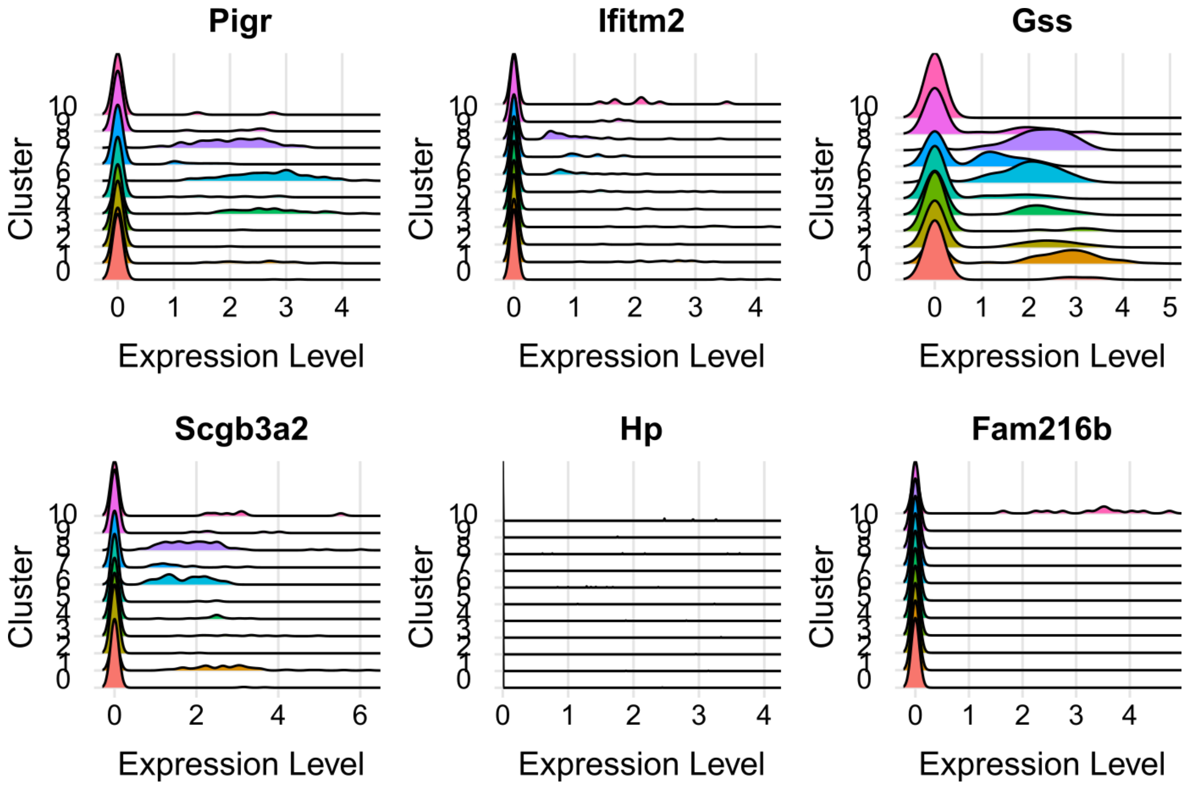

Supplementary figure 23: Ridge plots for club cell marker genes in SARS-CoV-2 infected hamsters. Results for club cell genes at 14 dpi. All genes were plotted individually. For club cell marker gene list, see Supplementary Table 1.

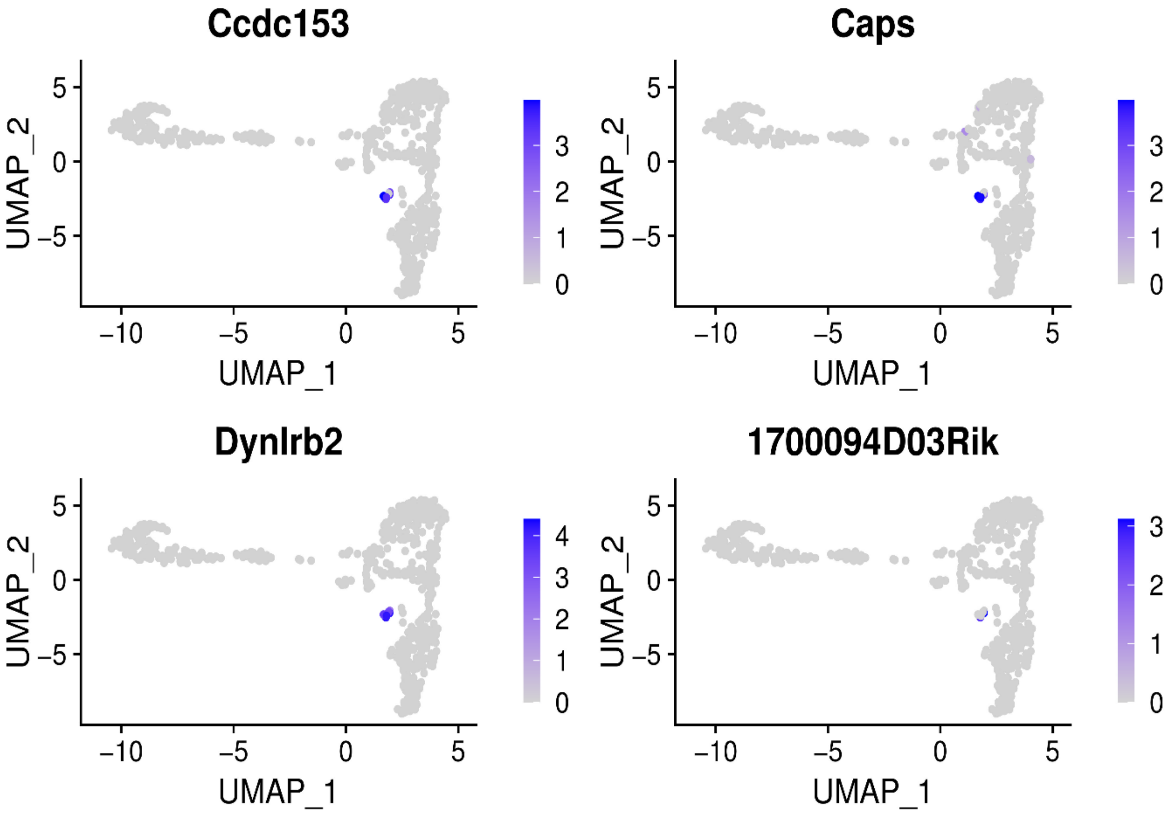

Supplementary figure 24: Feature plots for ciliated cell marker genes in SARS-CoV-2 infected hamsters. Results for ciliated cell genes at 5 dpi. All genes were plotted individually. For ciliated cell marker gene list, see Supplementary Table 1.

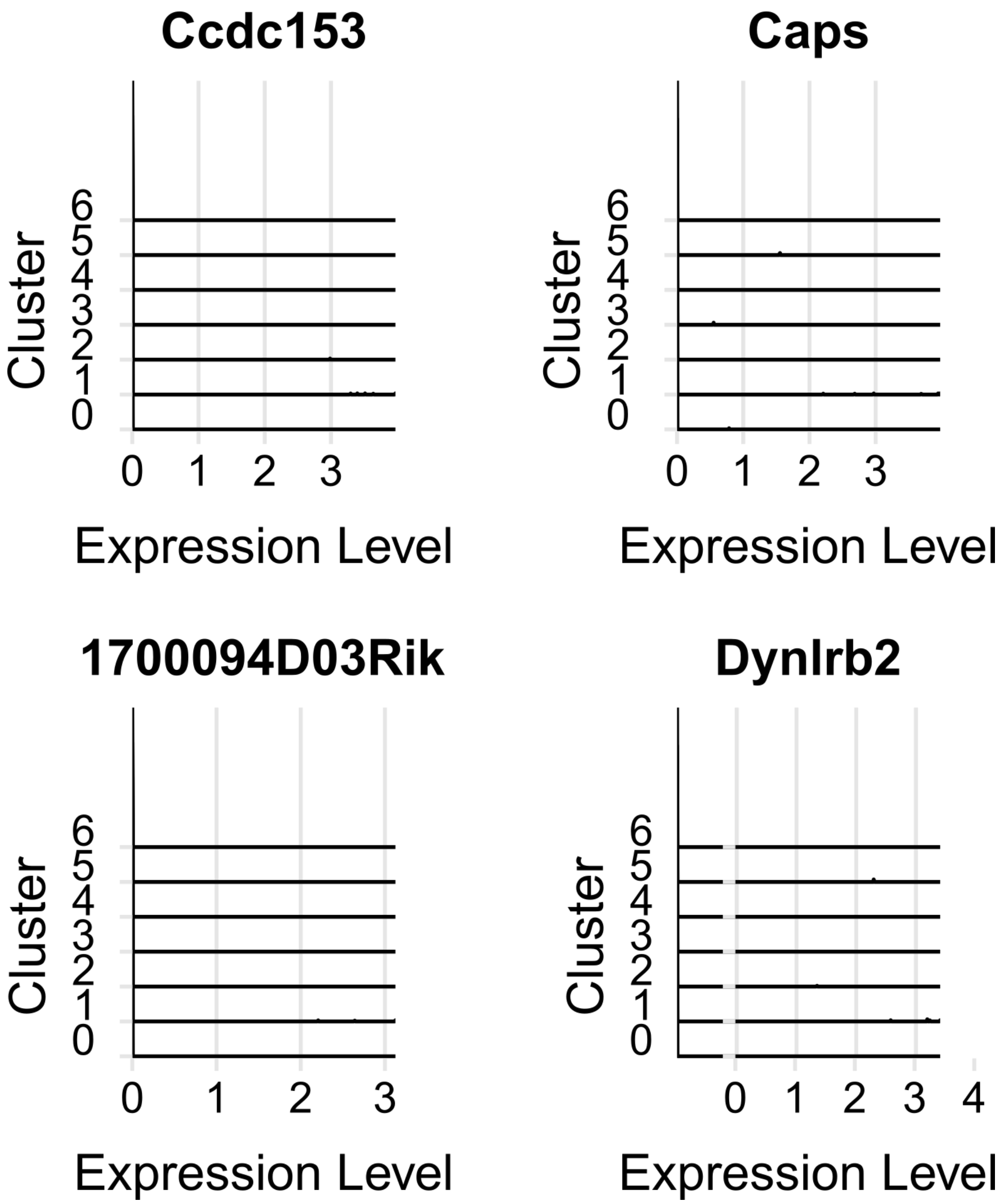

Supplementary figure 25: Ridge plots for ciliated cell marker genes in SARS-CoV-2 infected hamsters. Results for ciliated cell genes at 5 dpi. All genes were plotted individually. For ciliated cell marker gene list, see Supplementary Table 1.

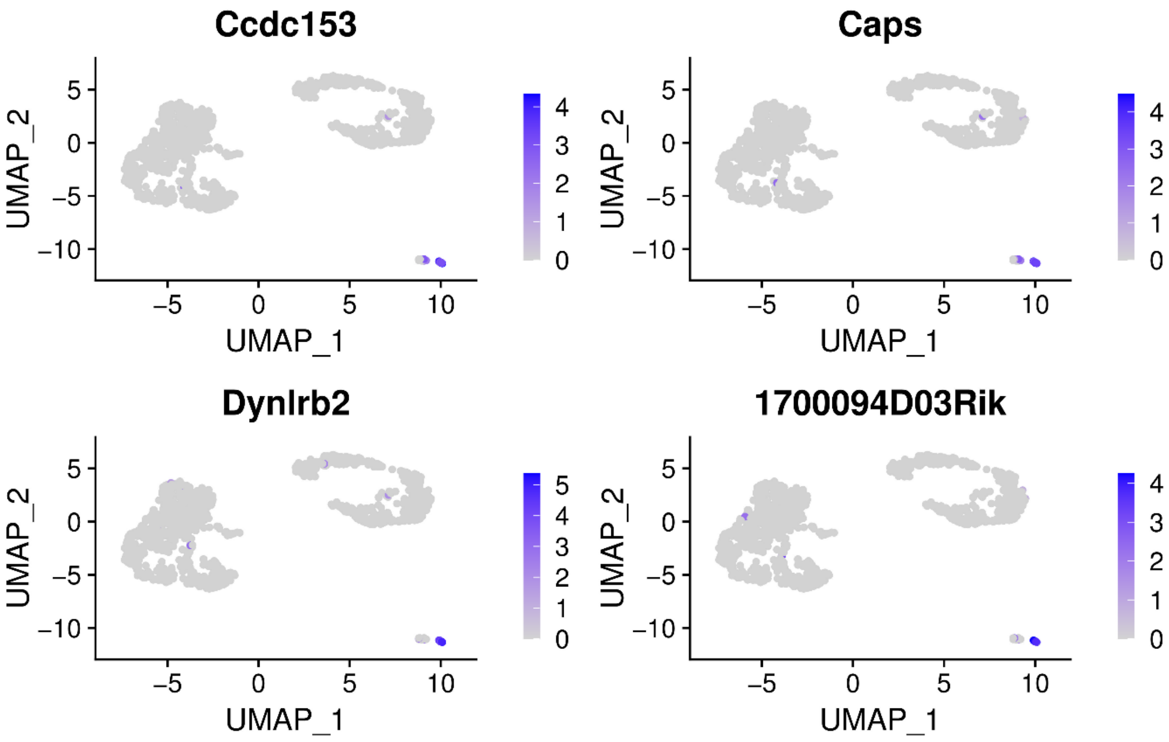

Supplementary figure 26: Feature plots for ciliated cell marker genes in SARS-CoV-2 infected hamsters. Results for ciliated cell genes at 14 dpi. All genes were plotted individually. For ciliated cell marker gene list, see Supplementary Table 1.

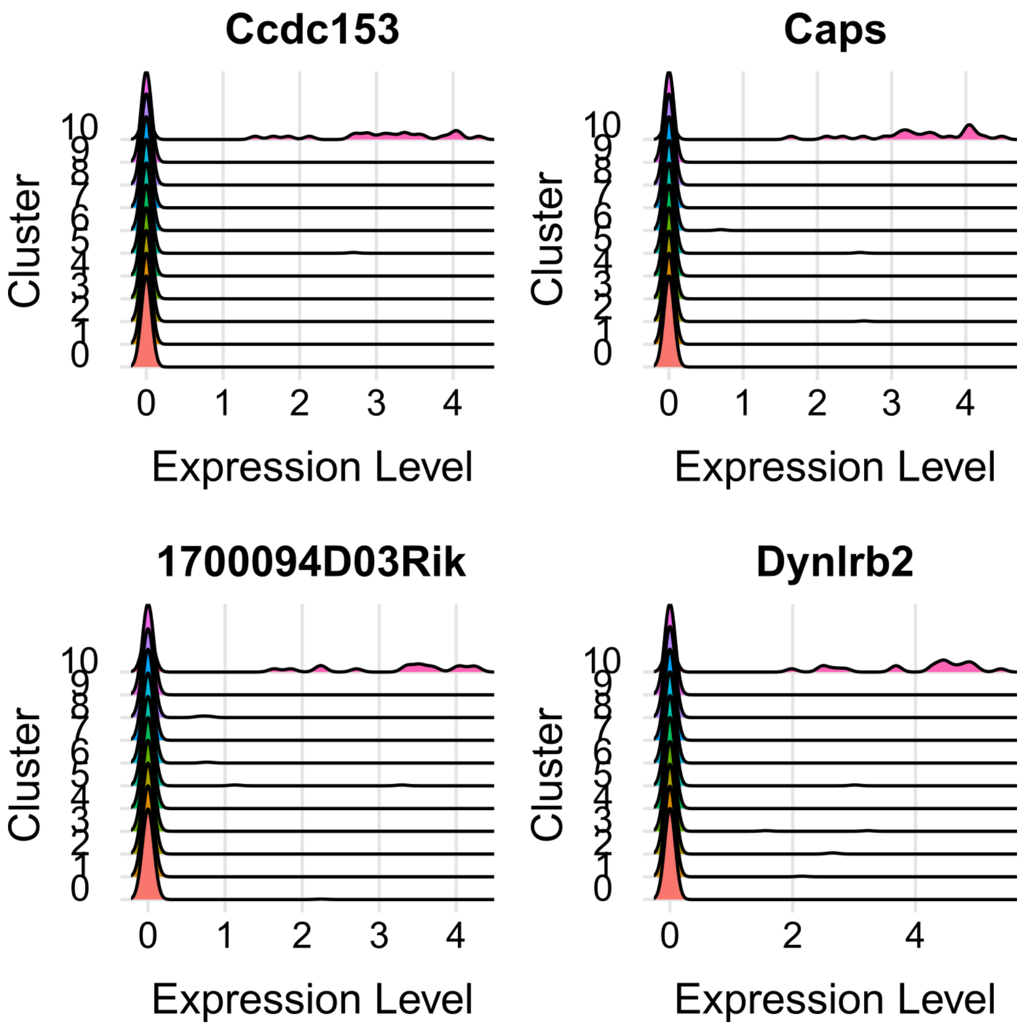

Supplementary figure 27: Ridge plots for ciliated cell marker genes in SARS-CoV-2 infected hamsters. Results for ciliated cell genes at 14 dpi. All genes were plotted individually. For ciliated cell marker gene list, see Supplementary Table 1.

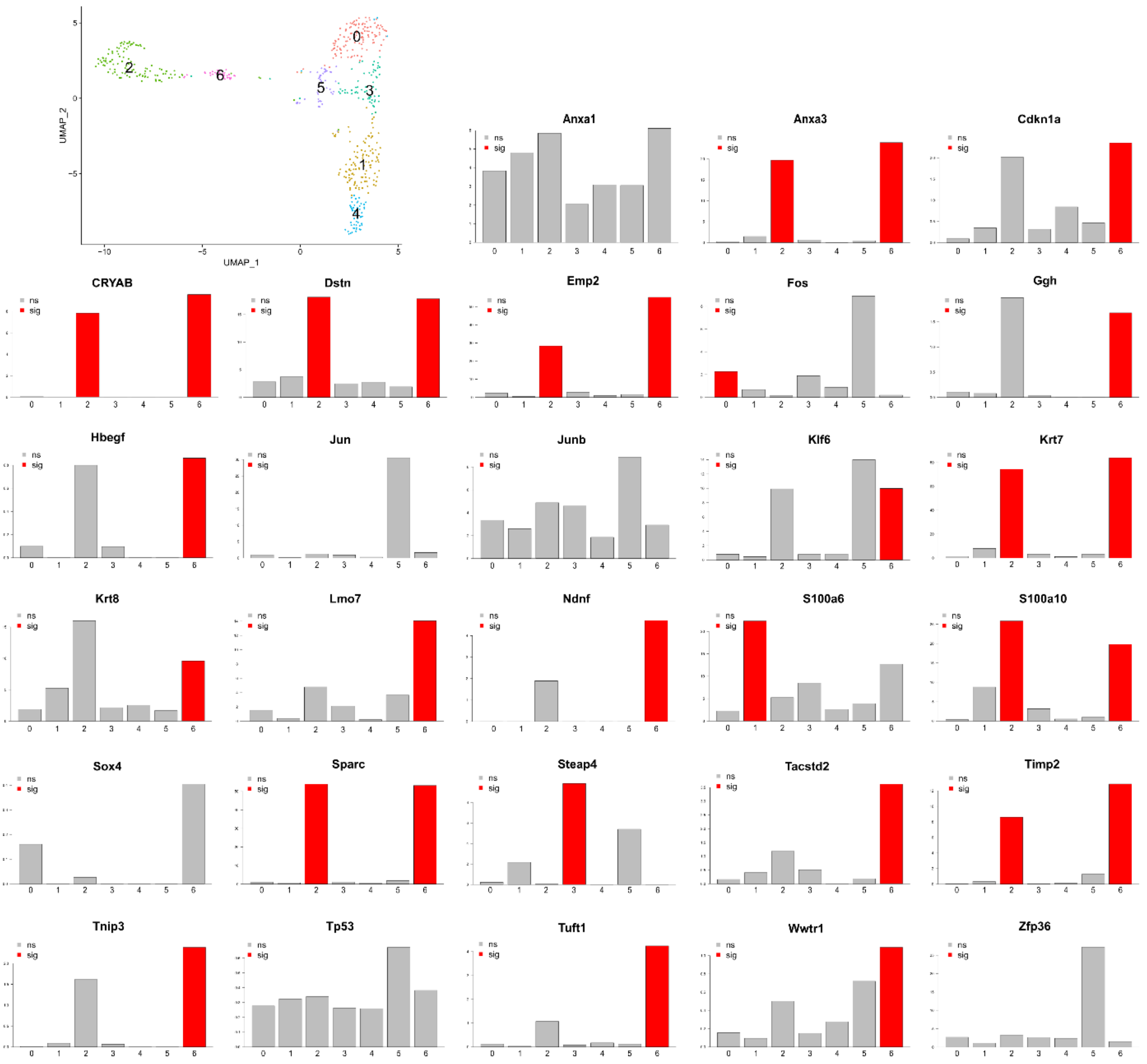

Supplementary figure 28: Mean expression levels of alveolar differentiation intermediate (ADI) cell genes in SARS-CoV-2 infected hamsters. Data were obtained from a published single cell RNA-Seq data set (GSE162208) from lungs of SARS-CoV-2 infected hamsters at 5 dpi. Alveolar cells were selected and re-clustered. Bar plots show ADI cell marker gene expression levels among clusters at 5 dpi. All genes were plotted individually. Bar plots indicate mean expression, red: significantly different mean gene expression (multiple testing adjusted p value <0.05); grey: not significantly different mean gene expression, y axis: mean gene expression level; x axis: cluster number. For individual ADI cell mean expression levels within clusters, see Supplementary Data 3, 4, and 5.

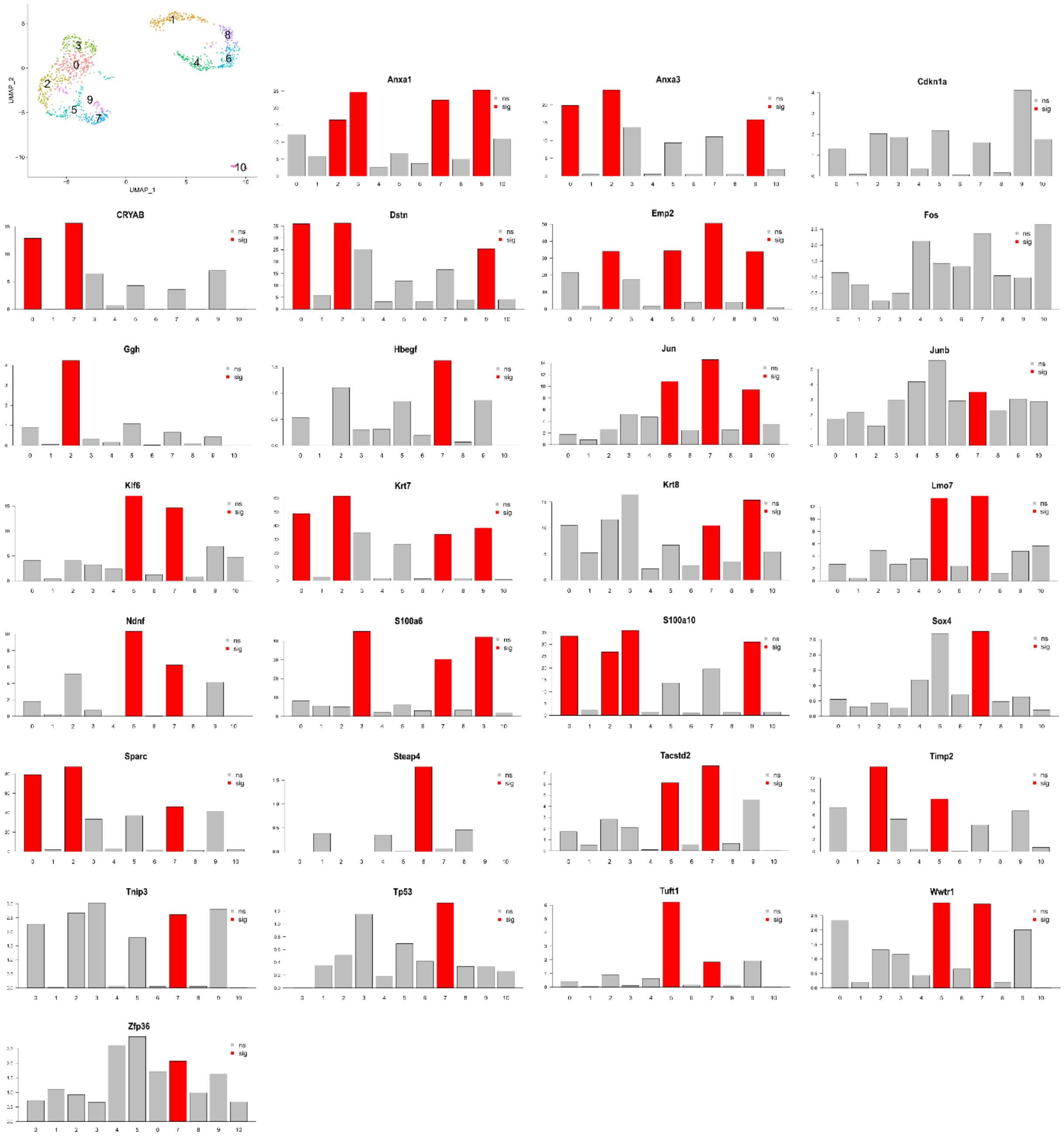

Supplementary figure 29: Mean expression levels of alveolar differentiation intermediate (ADI) cell genes in SARS-CoV-2 infected hamsters. Data were obtained from a published single cell RNA-Seq data set (GSE162208) from lungs of SARS-CoV-2 infected hamsters at 14 dpi. Alveolar cells were selected and re-clustered. Bar plots show ADI cell marker gene expression levels among clusters at 14 dpi. All genes were plotted individually. Bar plots indicate mean expression, red: significantly different mean gene expression (multiple testing adjusted p value <0.05); grey: not significantly different mean gene expression, y axis: mean gene expression level; x axis: cluster number. For individual ADI cell mean expression levels within clusters, see Supplementary Data 3, 4, and 5.
