# Supplementary material for: Hamster model for post-COVID-19 alveolar regeneration offers an opportunity to understand post-acute sequelae of SARS-CoV-2
Source: Nat Commun. 2023 Jun 5;14:3267. doi: 10.1038/s41467-023-39049-5 (PMC10241385; doi:10.1038/s41467-023-39049-5)
Supplement: Supplementary file 3 — Description of Additional Supplementary Files [file 41467_2023_39049_MOESM3_ESM.pdf]

Supplementary Data 1: list of differentially expressed genes (DEGs) per every cluster at 5 dpi showing gene name, average expression levels per cluster, multiple-testing adjusted p-values and fold changes.

This table is submitted as separate excel file.

Supplementary Data 2: list of differentially expressed genes (DEGs) per every cluster at 14 dpi showing gene name, average expression levels per cluster, multiple-testing adjusted p-values and fold changes.

This table is submitted as separate excel file.

Supplementary Data 3: Overview of ADI cells gene expression within clusters.

This table is submitted as separate excel file.

Supplementary Data 4: average expression of single ADI genes per cluster at 5 dpi.

This table is submitted as separate excel file.

Supplementary Data 5: average expression of single ADI genes per cluster at 14 dpi.

This table is submitted as separate excel file.
