# Supplementary material for: Hamster model for post-COVID-19 alveolar regeneration offers an opportunity to understand post-acute sequelae of SARS-CoV-2
Source: Nat Commun. 2023 Jun 5;14:3267. doi: 10.1038/s41467-023-39049-5 (PMC10241385; doi:10.1038/s41467-023-39049-5)
Supplement: Supplementary file 9 — Reporting Summary [file 41467_2023_39049_MOESM9_ESM.pdf]

nature portfolio

Corresponding author(s): Wolfgang Baumgärtner

Last updated by author(s): May 22, 2023

Reporting Summary

Nature Portfolio wishes to improve the reproducibility of the work that we publish. This form provides structure for consistency and transparency in reporting. For further information on Nature Portfolio policies, see our Editorial Policies and the Editorial Policy Checklist.

Statistics

For all statistical analyses, confirm that the following items are present in the figure legend, table legend, main text, or Methods section.

n/a	Confirmed	
☐	☒	The exact sample size (*n*) for each experimental group/condition, given as a discrete number and unit of measurement
☐	☒	A statement on whether measurements were taken from distinct samples or whether the same sample was measured repeatedly
☐	☒	The statistical test(s) used AND whether they are one- or two-sided<br>*Only common tests should be described solely by name; describe more complex techniques in the Methods section.*
☒	☐	A description of all covariates tested
☐	☒	A description of any assumptions or corrections, such as tests of normality and adjustment for multiple comparisons
☐	☒	A full description of the statistical parameters including central tendency (e.g. means) or other basic estimates (e.g. regression coefficient) AND variation (e.g. standard deviation) or associated estimates of uncertainty (e.g. confidence intervals)
☐	☒	For null hypothesis testing, the test statistic (e.g. *F*, *t*, *r*) with confidence intervals, effect sizes, degrees of freedom and *P* value noted<br>*Give P values as exact values whenever suitable.*
☒	☐	For Bayesian analysis, information on the choice of priors and Markov chain Monte Carlo settings
☒	☐	For hierarchical and complex designs, identification of the appropriate level for tests and full reporting of outcomes
☒	☐	Estimates of effect sizes (e.g. Cohen's *d*, Pearson's *r*), indicating how they were calculated

Our web collection on statistics for biologists contains articles on many of the points above.

Software and code

Policy information about availability of computer code

Data collection

No software was used for data collection.

Data analysis

scRNAseq data analysis: No custom software or codes were used. Data were analyzed using the R software package (version 3.6.0). The R package Seurat (version 3.2.0) was used for all analyses; details for Seurat functions used are provided in Material and Methods.

Statistical Analysis: GraphPad Prism Version 9.3.1

Image analysis: was performed using the open source software package QuPath (version 0.3.1) for digital pathology image analysis doi:10.1038/s41598-017-17204-5 (2017). For automatisation of tissue detection public available code was used to remove detected artifacts (dust particals and bubble) from tissue annotations .
URL: https://forum.image.sc/t/using-annotations-to-subtract-from-parent-object/25469/5
Code:

```
import qupath.lib.roi.*
import qupath.lib.objects.*

classToSubtract = null

def topLevel = getObjects{return it.getLevel()==1 && it.isAnnotation()}
println(topLevel)
for (parent in topLevel){
```

```
    def total = []
    def polygons = []
    subtractions = parent.getChildObjects().findAll{it.isAnnotation() }
    println(subtractions)

    for (subtractyBit in subtractions){
        if (subtractyBit instanceof AreaROI){
          subtractionROIs = PathROIToolsAwt.splitAreaToPolygons(subtractyBit.getROI())
          total.addAll(subtractionROIs[1])
        } else {total.addAll(subtractyBit.getROI())}

    }
    if (parent instanceof AreaROI){
        polygons = PathROIToolsAwt.splitAreaToPolygons(parent.getROI())
        total.addAll(polygons[0])
    } else { polygons[1] = parent.getROI()}


    def newPolygons = polygons[1].collect {
    updated = it
    for (hole in total)
         updated = PathROIToolsAwt.combineROIs(updated, hole, PathROIToolsAwt.CombineOp.SUBTRACT)
         return updated
    }
              // Remove original annotation, add new ones
    annotations = newPolygons.collect {new PathAnnotationObject(updated, parent.getPathClass())}


    addObjects(annotations)

    removeObjects(subtractions, true)
    removeObject(parent, true)
}
print "done"
```

To substract annotated areas from each other, multiple scripts where used.
To copy annotations within a project:

```
def path = buildFilePath(PROJECT_BASE_DIR, 'annotations')
def annotations = getAnnotationObjects()
new File(path).withObjectOutputStream {
    it.writeObject(annotations)
}
print 'Done!'
```

To paste annotations within a project:

```
def path = buildFilePath(PROJECT_BASE_DIR, 'annotations')
def annotations = null
new File(path).withObjectInputStream {
    annotations = it.readObject()
}
addObjects(annotations)
print 'Added ' + annotations
```

To substract one group of annotations from another (example):

```
tissueAnnotation = getAnnotationObjects().find{it.getPathClass() == getPathClass("Glandlike Proliferation")}
tissueGeom = tissueAnnotation.getROI().getGeometry()

//Cycle through ALL OTHER annotations and subtract them from the tissue

getAnnotationObjects().findAll{it.getPathClass() != getPathClass("Glandlike Proliferation")}.each{anno->
    currentGeom = anno.getROI().getGeometry()
    //Note the ! which means we are looking for NOT intersects
    tissueGeom = tissueGeom.difference(currentGeom)
}

//Create the new object
tissueROI = GeometryTools.geometryToROI(tissueGeom, ImagePlane.getDefaultPlane())
newTissue = PathObjects.createAnnotationObject( tissueROI, getPathClass("C") )
addObject(newTissue)

fireHierarchyUpdate()

def firstAnnotation = getAnnotationObjects().findAll{it.getPathClass() == getPathClass("Glandlike Proliferation")}
```

```
removeObjects(firstAnnotation, true)

To merge single annotations of the same group together:
selectObjectsByClassification("Airway");
mergeSelectedAnnotations()

selectObjectsByClassification("Blood Vessels");
mergeSelectedAnnotations()

selectObjectsByClassification("Glandlike Proliferation");
mergeSelectedAnnotations()

selectObjectsByClassification("Inflammation");
mergeSelectedAnnotations()

selectObjectsByClassification("Artifacts");
mergeSelectedAnnotations()

selectObjectsByClassification("Alveoli");
mergeSelectedAnnotations()

selectObjectsByClassification("Tissue");
mergeSelectedAnnotations()
```

For manuscripts utilizing custom algorithms or software that are central to the research but not yet described in published literature, software must be made available to editors and reviewers. We strongly encourage code deposition in a community repository (e.g. GitHub). See the Nature Portfolio guidelines for submitting code & software for further information.

Data

Policy information about availability of data

All manuscripts must include a data availability statement. This statement should provide the following information, where applicable:

- Accession codes, unique identifiers, or web links for publicly available datasets
- A description of any restrictions on data availability
- For clinical datasets or third party data, please ensure that the statement adheres to our policy

Source data are provided with this paper.
Single-cell RNASeq data from lungs of SARS-CoV-2 infected hamsters was obtained from a publicly available dataset; reference from GEO is provided: GSE162208.

Human research participants

Policy information about studies involving human research participants and Sex and Gender in Research.

Reporting on sex and gender	Lung samples from 3 male and one female patients were used (male/female refers to sex, gender information was not available. Sex was not considered in the selection process). No quantitative analysis was performed with human samples and therefore, no source data or sex-based analysis is provided.
Population characteristics	Lung samples were obtained from three patients who died of respiratory failure caused by severe COVID-19. The patients were two men, aged 76 and 74 years, and one woman, aged 74 years. The patients were hospitalized for 21, 7 and 5 days, respectively, and all received mechanical ventilation. SARS-CoV-2 infection was confirmed by PCR. The lung samples were obtained during autopsy. In addition, one non-COVID-19 lung sample was obtained from a 66-year-old man who underwent a lobectomy due to a pulmonary neoplasm.
Recruitment	Lung samples were selected based on the clinical and histopathological diagnosis (COVID-19 pneumonia and non-COVID-19 control). No potential self-selection bias or other biases impacted the results (only qualitative analysis performed on these samples).
Ethics oversight	Ethical approval was given by the local institutional review board at Hannover Medical School (no. 9621_BO_K_2021).

Note that full information on the approval of the study protocol must also be provided in the manuscript.

Field-specific reporting

Please select the one below that is the best fit for your research. If you are not sure, read the appropriate sections before making your selection.

☒ Life sciences ☐ Behavioural & social sciences ☐ Ecological, evolutionary & environmental sciences

For a reference copy of the document with all sections, see nature.com/documents/nr-reporting-summary-flat.pdf

Life sciences study design

All studies must disclose on these points even when the disclosure is negative.

Sample size	ANOVA and Kruskal-Wallis-Test were used to predetermine sample size
Data exclusions	All obtained data were included
Replication	The animal experiment, immunolabellings, quantifications and data analysis were performed once.
Randomization	Allocation to experimental groups was randomized.
Blinding	The investigators were not blinded to group allocation during the animal experiment for obvious reasons due to BSL3 safety conditions in order to avoid cross-infections between mock and SARS-CoV-2 infected animals. For morphologic analysis, samples were blinded before examination.

Reporting for specific materials, systems and methods

We require information from authors about some types of materials, experimental systems and methods used in many studies. Here, indicate whether each material, system or method listed is relevant to your study. If you are not sure if a list item applies to your research, read the appropriate section before selecting a response.

Materials & experimental systems

n/a	Involved in the study	
☐	☒	Antibodies
☐	☒	Eukaryotic cell lines
☒	☐	Palaeontology and archaeology
☐	☒	Animals and other organisms
☒	☐	Clinical data
☒	☐	Dual use research of concern

Methods

n/a	Involved in the study	
☒	☐	ChIP-seq
☒	☐	Flow cytometry
☒	☐	MRI-based neuroimaging

Antibodies

Antibodies used

Anti-SARS-CoV-2 NP (SinoBiological, 40143-MM05); Monoclonal Mouse IgG1 Clone #05
CK8 (Invitrogen, PA5-29607); Polyclonal rabbit
CK14 (Invitrogen, PA5-16722); Polyclonal rabbit
SCGB1A1 (Proteintec,10490-1-AP); Polyclonal rabbit
proSP-C (MEMD Millipore, AB3786); Polyclonal rabbit
IBA-1 (FUJIFILM Wako Pure Chemical Corporation, 019–19741); Polyclonal rabbit
α-SMA (Dako, GA611); Monoclonal mouse Clone 1A4
CD-204 (Abnova Corporation, MAB1710); Monoclonal mouse clone SRA-E5
CK8-FITC conjugated (abcam, ab192467); Monoclonal rabbit clone EP1628Y
CK14 (Invitrogen, PA5-16722); Polyclonal rabbit
CK14 (Invitrogen, MA5-11599); Monoclonal mouse clone LL002
TP53 (Novusbio, NBP2-29453); Monoclonal mouse clone BP53-12
ΔNp63 (Cell signalling, #67825S); Monoclonal rabbit clone E6Q30
CK5-FITC conjugated (Abcam, ab-193894); Monoclonal rabbit clone EP1601Y

Validation

All antibodies were used following the manufacturer's recommendations. Antibodies were validated for at least immunohistochemistry, immunofluorescence or immunoblotting by the manufacturer. Hamster species reactivity was not ensured for all antibodies. All antibodies ensured reactivity for Mice and Humans proteins. For this reason, before the experiment hamster reactivity was tested labelling different target organs for the investigated protein using mouse and hamster tissues in parallel and confirming antibodies specificity for the target.

Eukaryotic cell lines

Policy information about cell lines and Sex and Gender in Research

Cell line source(s)	VERO C1008 (ATCCR, CRL-1586)
Authentication	All cell lines used were purchased as stated above but not independently authenticated

Mycoplasma contamination	All cell lines used were mycoplasma free
Commonly misidentified lines (See ICLAC register)	no commonly misidentified cell lines were used.

Animals and other research organisms

Policy information about studies involving animals; ARRIVE guidelines recommended for reporting animal research, and Sex and Gender in Research

Laboratory animals	syrian golden hamsters (8-10 weeks)
Wild animals	The study did not involve wild animals
Reporting on sex	For this work, no sex- and gender-based analysis have been performed. Since only 5 male and 5 female hamsters were present in every group, these low numbers would not have allowed a reliable statistical analysis. For this reason, both sexes were pooled.
Field-collected samples	The study did not involve field-collected samples
Ethics oversight	The animal experiment was in accordance with the EU directive 2010/63/EU and approved Behörde für Justiz und Verbraucherschutz der Freien und Hansestadt Hamburg, Department for Lebensmittelsicherheit und Veterinärwesen (protocol code N032/2020 22 April 2020)

Note that full information on the approval of the study protocol must also be provided in the manuscript.
